# Recounting the FANTOM CAGE-Associated Transcriptome

**DOI:** 10.1101/gr.254656.119

**Published:** 2020-07

**Authors:** Eddie Luidy Imada, Diego Fernando Sanchez, Leonardo Collado-Torres, Christopher Wilks, Tejasvi Matam, Wikum Dinalankara, Aleksey Stupnikov, Francisco Lobo-Pereira, Chi-Wai Yip, Kayoko Yasuzawa, Naoto Kondo, Masayoshi Itoh, Harukazu Suzuki, Takeya Kasukawa, Chung-Chau Hon, Michiel J.L. de Hoon, Jay W. Shin, Piero Carninci, Andrew E. Jaffe, Jeffrey T. Leek, Alexander Favorov, Gloria R. Franco, Ben Langmead, Luigi Marchionni

**Affiliations:** 1 Department of Oncology, Johns Hopkins University School of Medicine, Baltimore, Maryland 21827, USA;; 2 Departamento de Bioqúımica e Imunologia, ICB, Universidade Federal de Minas Gerais, Belo Horizonte, Minas Gerais, 31270-901, Brazil;; 3 Lieber Institute for Brain Development, Baltimore, Maryland 21205, USA;; 4 Department of Computer Science, Johns Hopkins University, Baltimore, Maryland 21218, USA;; 5 Departamento de Biologia General, ICB, Universidade Federal de Minas Gerais, Belo Horizonte, Minas Gerais, 31270-901, Brazil;; 6 RIKEN Center for Integrative Medical Sciences, Yokohama, 230-0045, Japan;; 7 RIKEN, Preventive Medicine and Diagnostic Innovation Program, Yokohama, 351-0198, Japan;; 8 Department of Mental Health, Johns Hopkins Bloomberg School of Public Health, Baltimore, Maryland 21205, USA;; 9 Department of Biostatistics, Johns Hopkins Bloomberg School of Public Health, Baltimore, Maryland 21205, USA;; 10 Laboratory of Systems Biology and Computational Genetics, VIGG RAS, 117971 Moscow, Russia

## Abstract

Long noncoding RNAs (lncRNAs) have emerged as key coordinators of biological and cellular processes. Characterizing lncRNA expression across cells and tissues is key to understanding their role in determining phenotypes, including human diseases. We present here FC-R2, a comprehensive expression atlas across a broadly defined human transcriptome, inclusive of over 109,000 coding and noncoding genes, as described in the FANTOM CAGE-Associated Transcriptome (FANTOM-CAT) study. This atlas greatly extends the gene annotation used in the original *recount2* resource. We demonstrate the utility of the FC-R2 atlas by reproducing key findings from published large studies and by generating new results across normal and diseased human samples. In particular, we (a) identify tissue-specific transcription profiles for distinct classes of coding and noncoding genes, (b) perform differential expression analysis across thirteen cancer types, identifying novel noncoding genes potentially involved in tumor pathogenesis and progression, and (c) confirm the prognostic value for several enhancer lncRNAs expression in cancer. Our resource is instrumental for the systematic molecular characterization of lncRNA by the FANTOM6 Consortium. In conclusion, comprised of over 70,000 samples, the FC-R2 atlas will empower other researchers to investigate functions and biological roles of both known coding genes and novel lncRNAs.

Long noncoding RNAs (lncRNAs) are commonly defined as transcripts longer than 200 nucleotides that are not translated into proteins. This definition is not based on their function, since lncRNAs are involved in distinct molecular processes and biological contexts not yet fully characterized (Batista and Chang 2013). Over the past few years, the importance of lncRNAs has clearly emerged, leading to an increasing focus on decoding the consequences of their modulation and studying their involvement in the regulation of key biological mechanisms during development, normal tissue and cellular homeostasis, and in disease (Esteller 2011; Batista and Chang 2013; Ling et al. 2015).

Given the emerging and previously underestimated importance of noncoding RNAs (ncRNAs), the FANTOM Consortium has initiated the systematic characterization of their biological function. Through the use of Cap Analysis of Gene Expression sequencing (CAGE-seq), combined with RNA-seq data from the public domain, the FANTOM Consortium released a comprehensive atlas of the human transcriptome, encompassing more accurate transcriptional start sites (TSSs) for coding and noncoding genes, including numerous novel long noncoding genes: the FANTOM CAGE-Associated Transcriptome (FANTOM-CAT) (Hon et al. 2017). We hypothesized that these lncRNAs can be measured in many RNA-seq data sets from the public domain and that they have been so far missed by the lack of a comprehensive gene annotation.

Although the systematic analysis of lncRNAs function is being addressed by the FANTOM Consortium in loss-of-function studies, increasing the detection rate of these transcripts combining different studies is difficult because of the heterogeneity of analytic methods employed. Current resources that apply uniform analytic methods to create expression summaries from public data do exist but can miss several lncRNAs because of their dependency on a preexisting gene annotation for creating the gene expression summaries (Tatlow and Piccolo 2016; Lachmann et al. 2018). We recently created *recount2* (Collado-Torres et al. 2017b), a collection of uniformly processed human RNA-seq data, wherein we summarized 4.4 trillion reads from over 70,000 human samples from the NCBI Sequence Read Archive (SRA), The Cancer Genome Atlas (TCGA) (The Cancer Genome Atlas Research Network et al. 2013), and the Genotype-Tissue Expression (GTEx) (The GTEx Consortium 2013) projects (Collado-Torres et al. 2017b). Importantly, *recount2* provides annotation-agnostic coverage files that allow requantification using a new annotation without having to reprocess the RNA-seq data.

Given the unique opportunity to access the latest results to the most comprehensive human transcriptome (the *FANTOM-CAT* project) and the *recount2* gene agnostic summaries, we addressed the previously described challenges, building a comprehensive atlas of coding and noncoding gene expression across the human genome: the FANTOM-CAT*/recount2* expression atlas (FC-R2 hereafter). Our resource contains expression profiles for 109,873 putative genes across over 70,000 samples, enabling an unparalleled resource for the analysis of the human coding and noncoding transcriptome.

## Results

### Building the FANTOM-CAT/*recount2* resource

**Figure 1.**
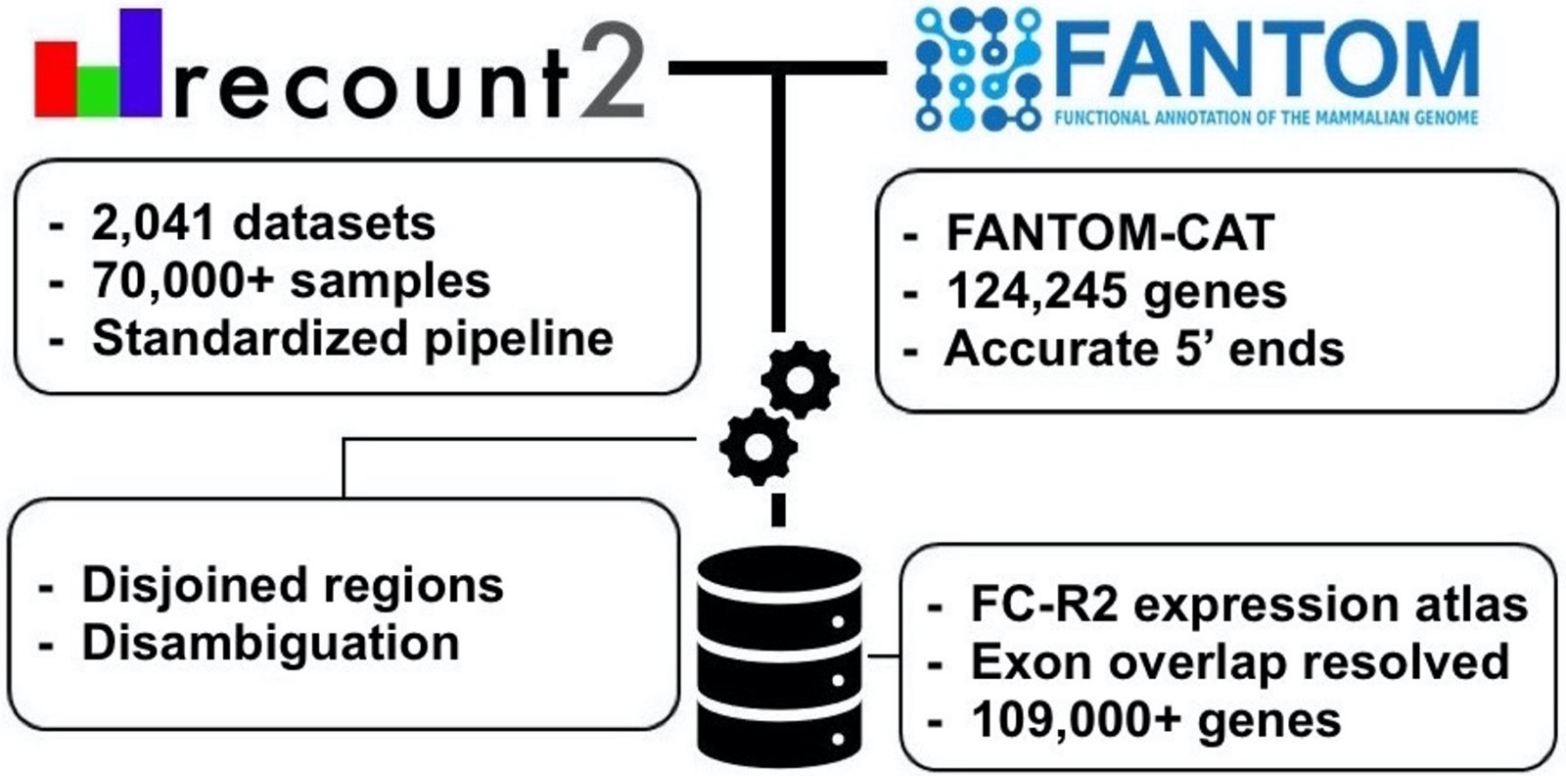
Overview of the FANTOM-CAT*/recount2* resource development. FC-R2 leverages two public resources, the FANTOM-CAT gene models and *recount2*. FC-R2 provides expression information for 109,873 genes, both coding (22,110) and noncoding (87,693). This latter group encompasses enhancers, promoters, and other lncRNAs.

The *recount2* resource includes a coverage track, in the form of a bigWig file, for each processed sample. We built the FC-R2 expression atlas by extracting expression levels from *recount2* coverage tracks in regions that overlapped unambiguous exon coordinates for the permissive set of FANTOM-CAT transcripts, according to the pipeline shown in Figure 1. Since *recount2*’s coverage tracks do not distinguish between genomic strands, we removed ambiguous segments that presented overlapping exon annotations from both strands (see Methods section and Supplemental Methods). After this disambiguation procedure, the remaining 1,066,515 exonic segments mapped back to 109,869 genes in FANTOM-CAT (out of the 124,047 starting ones included in the permissive set [Hon et al. 2017]). Overall, the FC-R2 expression atlas encompasses 2041 studies with 71,045 RNA-seq samples, providing expression information for 22,116 coding genes and 87,763 noncoding genes, such as enhancers, promoters, and other lncRNAs.

### Validating the *FANTOM*‐*CAT/recount2* resource

We first assessed how gene expression estimates in FC-R2 compared to previous gene expression estimates from other projects. Specifically, we considered data from the GTEx Consortium (v6), spanning 9662 samples from 551 individuals and 54 tissues types (The GTEx Consortium 2013). First, we computed the correlation for the GTEx data between gene expression based on the FC-R2 atlas and on the GENCODE (v25) gene model in *recount2*, which has been already shown to be consistent with gene expression estimates from the GTEx project (Collado-Torres et al. 2017b), observing a median correlation ≥0.986 for the 32,922 genes in common. This result supports the notion that our preprocessing steps to disambiguate overlapping exon regions between strands did not significantly alter gene expression quantification.

**Figure 2.**
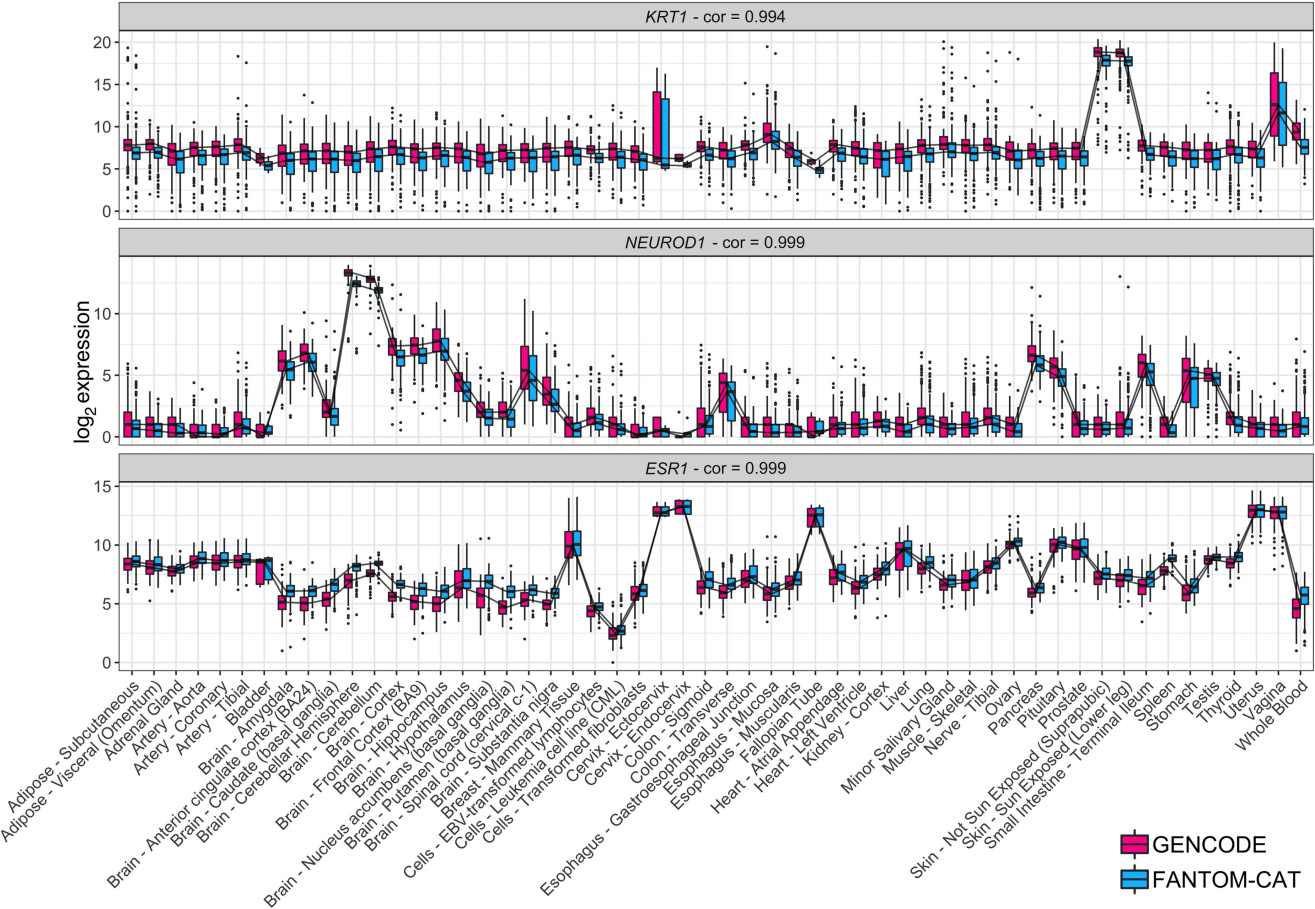
Tissue-specific expression in GTEx. Log_2_ expression for three tissue-specific genes (*KRT1*, *NEUROD1*, and *ESR1*) in GTEx data stratified by tissue type using FC-R2- and GENCODE-based quantification. Expression profiles are highly correlated and expressed consistently in the expected tissue types (e.g., *KRT1* is most expressed in skin, *NEUROD1* in brain, and *ESR1* in estrogen-sensitive tissue types like uterus, Fallopian tubes, and breast). Correlations are shown on *top* for each tissue marker. *Center* lines, *upper*/*lower* quartiles, and whiskers represent the median, 25/75 percentiles, and 1.5 interquartile range, respectively. Additional tissue-specific markers are shown in Supplemental Figure S1.

Next, we assessed whether gene expression specificity, as measured in FC-R2, was maintained across tissue types. To this end, we selected and compared gene expression for known tissue-specific expression patterns, such as keratin 1 (*KRT1*), estrogen receptor 1 (*ESR1*), and neuronal differentiation 1 (*NEUROD1*) (Fig. 2). Overall, all analyzed tissue-specific markers presented nearly identical expression profiles across GTEx tissue types between the alternative gene models considered (see Fig. 2 and Supplemental Fig. S1), confirming the consistency between gene expression quantification in FC-R2 and those based on GENCODE.

We also assessed whether there are genes that are not expressed in any of the normal tissues included in GTEx. Out of 109,869 genes, 681 (0.6%) (see Supplemental Figs. S3, S4) were not expressed in any tissue included in GTEx, and they were overrepresented in the FANTOM-CAT permissive set (χ^2^ test, *P*-value < 2.2 × 10 ^16^).

### Tissue-specific expression of lncRNAs

**Figure 3.**
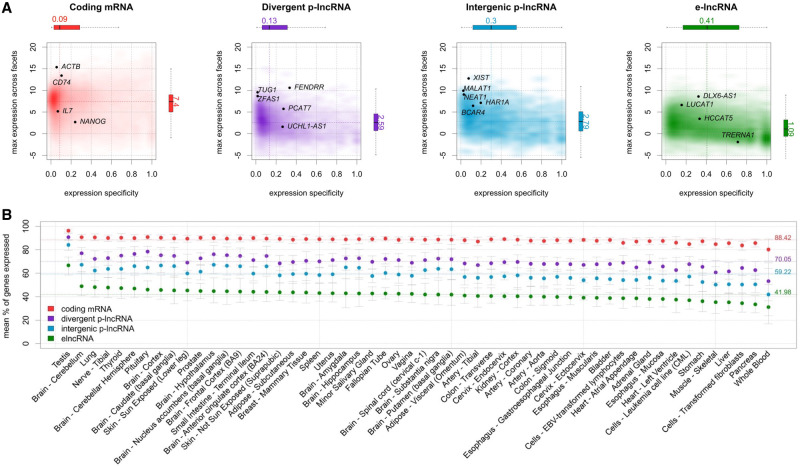
Expression profiles across GTEx tissues. (*A*) Expression level and tissue specificity across four distinct RNA categories. The *y*-axis shows log_2_ expression levels representing each gene using its maximum expression in GTEx tissues expressed as transcripts per million (TPM). The *x*-axis shows expression specificity based on entropy computed from median expression of each gene across the GTEx tissue types. Individual genes are highlighted in the figure panels. (*B*) Percentage of genes expressed for each RNA category stratified by GTEx tissue facets. The dots represent the mean among samples within a facet and the error bars represent 99.99% confidence intervals. Dashed lines represent the means among all samples.

It has been shown that, although expressed at a lower level, enhancers and promoters are not ubiquitously expressed and are more specific for different cell types than coding genes (Hon et al. 2017). In order to verify this finding, we used GTEx data to assess expression levels and specificity profiles across samples from each of the 54 analyzed tissue types, stratified into four distinct gene categories: coding mRNA, intergenic promoter lncRNA (ip-lncRNA), divergent promoter lncRNA (dp-lncRNA), and enhancer lncRNA (e-lncRNA). Overall, we were able to confirm that these RNA classes are expressed at different levels and that they display distinct specificity patterns across tissues, as shown for primary cell types by Hon et al. (2017), albeit with more variability, likely due to the increased cellular complexity present in tissues. Specifically, coding mRNAs were expressed at higher levels than lncRNAs (log_2_ median expression of 6.6 for coding mRNAs, and of 4.1, 3.8, and 3.1 for ip-lncRNA, dp-lncRNA, and e-lncRNA, respectively). In contrast, the expression of enhancers and intergenic promoters was more tissue-specific (median = 0.41 and 0.30, respectively) than that observed for divergent promoters and coding mRNAs (median = 0.13 and 0.09, respectively) (Fig. 3A). Finally, when analyzing the percentage of genes expressed across tissues by category, we observed that coding genes are, in general, more ubiquitous, whereas lncRNAs are more specific, with enhancers showing the lowest percentages of expressed genes (mean ranging from 88.42% to 41.98%) (see Fig. 3B), in agreement with the notion that enhancer transcription is tissue-specific (Ong and Corces 2011).

### Differential expression analysis of coding and noncoding genes in cancer

**Figure 4.**
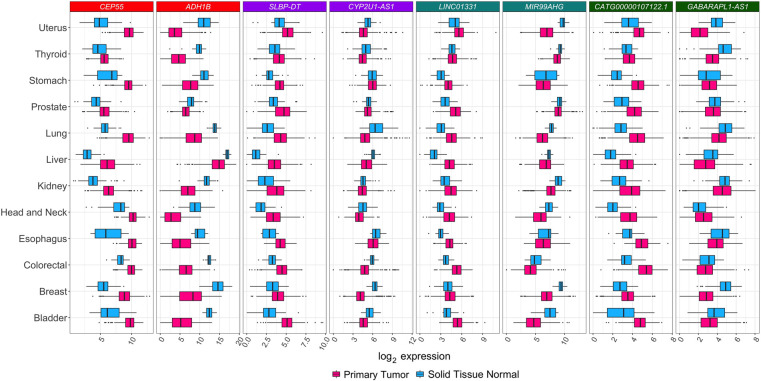
Differential expression for selected transcripts from distinct RNA classes across tumor types. Box plots for selected differentially expressed genes between tumor and normal samples across all 13 tumor types analyzed. For each tissue of origin, the most up-regulated (on the *left*) and down-regulated (on the *right*) gene for each RNA class is shown. *Center* lines, *upper*/*lower* hinges, and the whiskers, respectively, represent the median, the upper and lower quartiles, and 1.5 extensions of the interquartile range. Color coding on the *top* of the figure indicates the RNA classes (red for mRNA, purple for dp-lncRNA, cyan ip-lncRNA, and green for e-lncRNA). These genes were selected after global multiple testing correction across all 13 tumor types (see Supplemental Tables S1–S4).

We analyzed coding and noncoding gene expression in cancer using TCGA data. To this end, we compared cancer to normal samples separately for 13 tumor types, using FC-R2 requantified data. We further identified the differentially expressed genes (DEGs) in common across the distinct cancer types (see Fig. 4). Overall, the number of DEGs varied across cancer types and by gene class, with a higher number of significant coding than noncoding genes (FDR ≤ 0.01) (see Table 1). A substantial fraction of these genes was exclusively annotated in the FANTOM-CAT meta-assembly, suggesting that relying on other gene models would result in missing many potential important genes (see Table 1). We then analyzed differential gene expression consensus across the considered cancer types. A total of 41 coding mRNAs were differentially expressed across all of the 13 tumor types after global correction for multiple testing (FDR ≤ 10^−6^) (see Supplemental Table S1). For lncRNAs, a total of 28 divergent promoters, four intergenic promoters, and three enhancers were consistently up- or down-regulated across all the 13 tumor types after global correction for multiple testing (FDR ≤ 0.1) (see Supplemental Tables S2–S4, respectively).

**Table 1.**
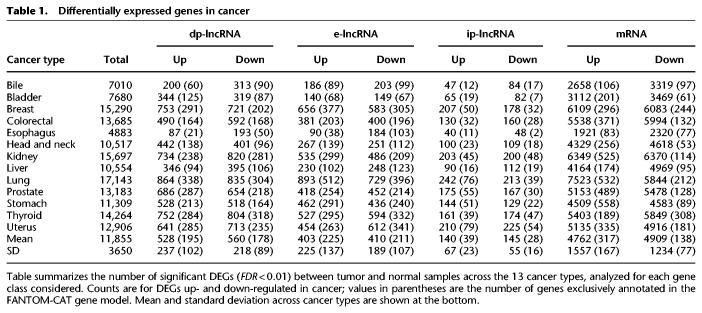
Differentially expressed genes in cancer

A usual task performed after differential gene expression analysis is to identify biological processes and pathways associated with the DEGs. To this end, gene set enrichment methods are usually employed; however, this requires detailed gene-to-function annotations, which are mostly lacking for lncRNAs. One possible way to assist prioritizing noncoding transcripts for follow-up functional studies is to identify association with other features along the genome. As an example of this type of analysis, we have assessed the overlap between single-nucleotide polymorphisms (SNPs) associated with cancer in GWAS studies and the list of DEGs we identified. On average, the percentage of DEGs overlapping cancer SNPs ranged from 6.6% in dp-lncRNA to 10.21% in ip-lncRNA across the 13 cancer types (see Supplemental Table S5).

Next, we reviewed the literature to identify functional correlates for these consensus genes. Most of the up-regulated coding genes (Supplemental Table S1) participate in cell cycle regulation, cell division, DNA replication and repair, chromosome segregation, and mitotic spindle checkpoints. Most of the consensus down-regulated mRNAs (Supplemental Table S1) are associated with metabolism and oxidative stress, transcriptional regulation, cell migration and adhesion, and with modulation of DNA damage repair and apoptosis.

Three down-regulated dp-lncRNA genes, *GAS1RR*, *RPL34-DT*, and *RAP2C-AS1*, were reported to be implicated in cancer (Supplemental Table S2). The first one controls epithelial-mesenchymal transition, the second is associated with tumor size increase, whereas the third is associated with urothelial cancer after kidney cancer transplantation (Zhao et al. 2015b; Shang et al. 2016; Zhou et al. 2016). Among the up-regulated dp-lncRNAs (Supplemental Table S2), *SNHG1* has been implicated in cellular proliferation and migration and invasion of different cancer types, and to be strongly up-regulated in osteosarcoma, nonsmall lung cancer, and gastric cancer (Cao et al. 2013; Sun et al. 2017).

Among the ip-lncRNAs ubiquitously down-regulated (see Supplemental Table S3), *MIR99AHG* has been identified in many different tumor types, including leukemia, breast, vulvar, prostate, and bladder cancer (Emmrich et al. 2014; Sun et al. 2014; Gökmen-Polar et al. 2016; Ni et al. 2016; Li et al. 2017). For instance, in vulvar squamous cell carcinoma, *MIR99AHG* and *MIR31HG* expressions are correlated and associated with tumor differentiation (Ni et al. 2016). Similarly, *MIR99AHG* down-regulation in ER-positive breast cancer is associated with progression, recurrence, and metastasis (Gökmen-Polar et al. 2016). In contrast, increased expression of *SNHG17* (an ip-lncRNA) (see Supplemental Table S3) was associated with short term survival in breast cancer and with tumor size, stage, and lymph node metastasis in colorectal cancer (Zhao et al. 2015a; Ma et al. 2017). In addition, *LINC01311*, another ip-lncRNA (Supplemental Table S3), was found to be up-regulated in liver cancer and metastatic prostate cancer (Zhu et al. 2016). Even though we did not identify any cancer association for common e-lncRNAs, one among those we identified, *LINC02884*, has been previously reported to be up-regulated in late-onset Alzheimer's disease (Humphries et al. 2015). Furthermore, the enhancer lncRNA class also yielded the lowest number of genes in common among all cancer types, reinforcing the concept that enhancers are expressed in a tissue-specific manner (see Fig. 3A and Supplemental Table S4).

Finally, we focused more in depth on prostate cancer (PCa) as a prototypical example, and we were able to confirm previous findings for both coding and noncoding genes (see Supplemental Fig. S2). For coding genes, we confirmed differential expression for known markers of PCa progression and mortality, like *ERG*, *FOXA1*, *RNASEL*, *ARVCF*, and *SLC43A1* (Yu et al. 2010; Lin et al. 2011). Similarly, we also confirmed differential expression for noncoding genes, like *PCA3*, the first clinically approved lncRNA marker for PCa (Bussemakers et al. 1999; de Kok et al. 2002), *PCAT1*, a prostate-specific lncRNA involved in disease progression (Prensner et al. 2011), *MALAT1*, which is associated with PCa poor prognosis (Ren et al. 2013), *CDKN2B-AS1*, an antisense lncRNA up-regulated in PCa that inhibits tumor suppressor genes activity (Kotake et al. 2011; Gutschner and Diederichs 2012), and the *MIR135* host gene, which is associated with castration-resistant PCa (Huang et al. 2015).

### Confirming prognostic enhancers

Chen and collaborators have recently surveyed enhancer expression in nearly 9000 patients from TCGA (Chen et al. 2018), using genomic coordinates from the FANTOM5 project (Andersson et al. 2014), identifying 4803 expressed genomic regions with prognostic potential in one or more TCGA tumor types. We therefore leveraged the FC-R2 atlas to identify prognostic coding and noncoding genes using both univariate and multivariate Cox proportional hazard models, comparing our results for e-lncRNAs with those reported by Chen and colleagues. To this end, we started by comparing gene annotations and genomic overlap between the studies. This was necessary because Chen and collaborators relied on the enhancer regions reported by Andersson et al. (2014), which is based on the observation of bidirectional transcription. Our resource, on the contrary, relies on the latest updated FANTOM-CAT annotation, which takes into account other features, such as the epigenetic context, when defining RNA categories. Out of the 4803 genomic regions found prognostic by Chen and collaborators (Chen et al. 2018), we could unambiguously map 1218 regions to exons annotated in the FANTOM-CAT gene models for the four RNA categories we considered in our study (corresponding to a total of 1046 unique genes). Overall, despite the mentioned differences in annotation and quantification (see Supplemental Table S6), we were still able to confirm the prognostic value for 466 genes out of the 1046 reported by Chen et al. (2018), including *KLHDC7B-DT* (also known as enhancer 22), which was highlighted as a promising prognostic marker for kidney cancer (Supplemental Fig. S5).

We then considered the FANTOM-CAT RNA classes across the different tumor types. We were able to identify a variable number of genes significantly associated with overall survival (FDR ≤ 0.05) in univariate Cox proportional hazards models (see Supplemental Tables S7–S10). Among the consensus DEGs identified across all tumor types, 40 out of 41 coding mRNAs, 25 out of 28 dp-lncRNAs, four out of four ip-lncRNAs, and two out of three e-lncRNAs were found to be associated with survival (see Supplemental Tables S11–S14). Kaplan–Meier curves for selected differentially expressed genes for each RNA category are shown in Supplemental Figure S6. Finally, we performed multivariable analysis controlling for relevant clinical and pathological characteristics in each tumor type. Overall, despite a number of genes being associated with such variables, we obtained similar results (see Supplemental Tables S15–S22).

## Discussion

The importance of lncRNAs in cell biology and disease has clearly emerged in the past few years, and different classes of lncRNAs have been shown to play crucial roles in cell regulation and homeostasis (Quinn and Chang 2016). For instance, enhancers—a major category of gene regulatory elements, which has been shown to be expressed (Andersson et al. 2014; Arner et al. 2015)—play a prominent role in oncogenic processes (Herz et al. 2014; Sur and Taipale 2016) and other human diseases (Hnisz et al. 2013). Despite their importance, however, there is a scarcity of large-scale data sets investigating enhancers and other lncRNA categories, in part due to the technical difficulty in applying high-throughput techniques such as ChIP-seq and Hi-C over large cohorts, and to the use of gene models that do not account for them in transcriptomics analyses. Furthermore, the large majority of the lncRNAs that are already known—and that have been shown to be associated with some phenotype—are still lacking functional annotation.

To address these needs, the FANTOM Consortium has first constructed the FANTOM-CAT metatranscriptome, a comprehensive atlas of coding and noncoding genes with robust support from CAGE-seq data (Hon et al. 2017); then, it has undertaken a large scale project to systematically target lncRNAs and characterize their function using a multipronged approach (Ramilowski et al. 2020). In a complementary effort, we have leveraged public domain gene expression data from *recount2* (Collado-Torres et al. 2017a,b) to create a comprehensive gene expression compendium across human cells and tissues based on the FANTOM-CAT gene model, with the ultimate goal of facilitating lncRNAs annotation through association studies. To this end, the FC-R2 atlas is already in use in the FANTOM6 project (https://fantom.gsc.riken.jp/6/) to successfully characterize lncRNA expression in human samples (Ramilowski et al. 2020).

In order to validate our resource, we have compared the gene expression summaries based on FANTOM-CAT gene models with previous, well-established gene expression quantifications, demonstrating virtually identical profiles across tissue types overall and for specific tissue markers. We have then confirmed that distinct classes of coding and noncoding genes differ in terms of overall expression level and specificity pattern across cell types and tissues. We also have observed a small subset of genes that were not expressed in the large majority of the samples analyzed in the GTEx project. These genes were mostly classified as small RNAs and enhancers, which was expected given that the RNA-seq libraries included in *recount2* did not target small RNAs, and enhancers are usually expressed at a lower level. We further reveal that this subset of genes not expressed in any normal tissue is also associated with a lower level of support of the corresponding FANTOM-CAT gene models (Hon et al. 2017).

Furthermore, using the FC-R2 atlas, we were also able to identify mRNAs, promoters, enhancers, and other lncRNAs that are differentially expressed in cancer, both confirming previously reported findings and identifying novel cancer genes exclusively annotated in the FANTOM-CAT gene models, which have been therefore missed in prior analyses with TCGA data. Finally, we confirmed the prognostic value for some of the enhancer regions recently reported by Chen and colleagues in the TCGA (Chen et al. 2018) by performing a systematic screening for survival association of both coding and noncoding genes that are quantifiable in the FC-R2 resource. Overall, we identified several genes with potential prognostic value across the analyzed cancer types in TCGA; however, further corroboratory studies in independent patient cohorts are necessary to validate these associations.

Collectively, by confirming findings reported in previous studies, our results demonstrate that the FC-R2 gene expression atlas is a reliable and powerful resource for exploring both the coding and noncoding transcriptome, providing compelling evidence and robust support to the notion that lncRNA gene classes, including enhancers and promoters, despite not being yet fully understood, portend significant biological functions. Our resource, therefore, constitutes a suitable and promising platform for future large scale studies in cancer and other human diseases, which in turn hold the potential to reveal important cues to the understanding of their biological, physiological, and pathological roles, potentially leading to improved diagnostic and therapeutic interventions.

Finally, all results, data, and code from the FC-R2 atlas are available as a public tool. With uniformly processed expression data for over 70,000 samples and 109,873 genes ready to analyze, we want to encourage researchers to dive deeper into the study of ncRNAs, their interaction with coding and noncoding genes, and their influence on normal and disease tissues. We hope this new resource will help pave the way to develop new hypotheses that can be followed to unwind the biological role of the transcriptome as a whole.

## Methods

### Data and preprocessing

**Figure 5.**
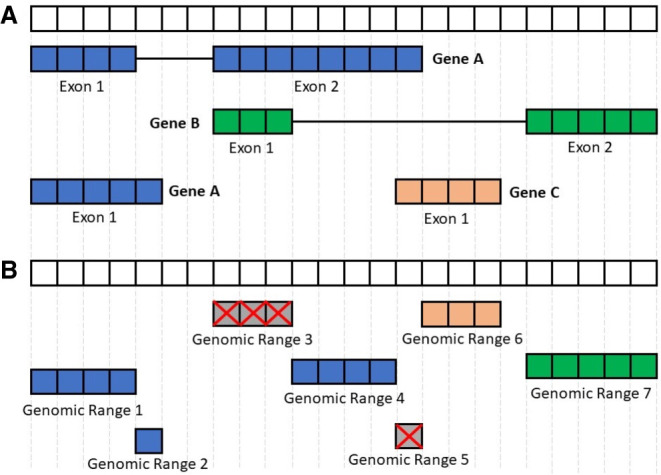
Processing the FANTOM-CAT genomic ranges. This figure summarizes the disjoining and exon disambiguation processes performed before extracting expression information from *recount2* using the FANTOM-CAT gene models. (*A*) Representation of a genomic segment containing three distinct, hypothetical genes: gene A having two isoforms, and genes B and C with one isoform each. Each box can be interpreted as one nucleotide along the genome. Colors indicate the three different genes. (*B*) Representation of disjoint exon ranges from example in panel *A*. Each feature is reduced to a set of nonoverlapping genomic ranges. The disjoint genomic ranges mapping back to two or more distinct genes are removed (crossed gray boxes). After removal of ambiguous ranges, the expression information for the remaining ones is extracted from *recount2* and summarized at the gene level.

The complete FANTOM-CAT gene catalog (inclusive of robust, intermediate, and permissive sets) was obtained from the FANTOM Consortium within the frame of the FANTOM6 project (Ramilowski et al. 2020). The genes were annotated using official HUGO Gene Nomenclature Committee (HGNC) symbols (https://www.genenames.org) when available. For genes without HGNC symbols, we named them according to HGNC instructions (see Supplemental Table S23). The remaining genes were referred to using the official ID from the Consortium that annotated the gene (Ensembl/FANTOM). This catalog accounts for 124,245 genes supported by CAGE peaks, and it includes those described by Hon et al. (2017). In order to remove ambiguity due to overlapping among exons from distinct genes, the BED files containing the coordinates for all genes and exons were processed with the *GenomicRanges* R/Bioconductor package (Lawrence et al. 2013) to obtain disjoint (nonoverlapping) exon coordinates. To avoid losing strand information from annotation, we processed data using a two-step approach by first disjoining overlapping segments on the same strand and then across strands (Fig. 5). The genomic ranges (disjoint exon segments) that mapped back to more than one gene were discarded. The expression values for these ranges were then quantified using *recount.bwtool* (Ellis et al. 2018) (code at https://github.com/LieberInstitute/marchionni_projects). The resulting expression quantifications were processed to generate RangedSummarizedExperiment objects compatible with the *recount2* framework (Collado-Torres et al. 2017a,b) (code available from https://github.com/eddieimada/fcr2). Thus, the FC-R2 atlas provides expression information for coding and noncoding genes (including enhancers, divergent promoters, and intergenic lncRNAs) for 9662 samples from the GTEx project, 11,350 samples from TCGA, and over 50,000 samples from the SRA.

### Correlation with other studies

To test if the preprocessing steps used for FC-R2 had a major impact on gene expression quantification, we compared our data to the published GTEx expression values obtained from *recount2* (version 2, https://jhubiostatistics.shinyapps.io/recount/). Specifically, we first compared the expression distribution of tissue-specific genes across different tissue types and then computed the Pearson's correlation for each gene in common across the original *recount2* gene expression estimates based on GENCODE and our version based on the FANTOM-CAT transcriptome.

### Expression specificity of tissue facets

We analyzed the expression level and specificity of each gene stratified by RNA category (i.e., mRNA, e-lncRNA, dp-lncRNA, ip-lncRNA) using the same approach described by Hon et al. (2017) (see Supplemental Methods). Briefly, overall expression levels for each gene were represented by the maximum transcript per million (TPM) values observed across all samples within each tissue type in GTEx. Gene specificity was based on the empirical entropy computed using the mean expression value across tissue types. The 99.99% confidence intervals for the expression of each category by tissue type were calculated based on TPM values. Genes with a TPM greater than 0.01 were considered to be expressed.

### Identification of differentially expressed genes

We analyzed differential gene expression in 13 cancer types, comparing primary tumor with normal samples using TCGA data from the FC-R2 atlas. Gene expression summaries for each cancer type were split by RNA category (coding mRNA, intergenic promoter lncRNA, divergent promoter lncRNA, and enhancer lncRNA) and then analyzed independently. A generalized linear model approach, coupled with empirical Bayes moderation of standard errors (Smyth 2004), was used to identify differentially expressed genes between groups. The model was adjusted for the three most relevant coefficients for data heterogeneity as estimated by surrogate variable analysis (SVA) (Leek and Storey 2007). Correction for multiple testing was performed across RNA category by merging the resulting *P*-values for each cancer type and applying the Benjamini–Hochberg method (Benjamini and Hochberg 1995). Overlapping between DEG and GWAS SNPs was performed using the FANTOM-CAT gene regions coordinates and the SNPs positions obtained from the GWAS catalog (Buniello et al. 2019).

### Prognostic analysis

To evaluate the prognostic potential of the genes in FC-R2, we performed both multivariate and univariate Cox proportional hazards regression analysis separately for each RNA class (22,106 mRNAs, 17,404 e-lncRNAs, 6204 dp-lncRNAs, and 1948 ip-lncRNAs) across each of the 13 TCGA cancer types with available survival follow-up information (see Supplemental Methods; Supplemental Table S24). Genes with FDR ≤ 0.05, using the Benjamini–Hochberg correction (Benjamini and Hochberg 1995) within each cancer type and RNA class, were deemed significant prognostic factors. We further analyzed the prognostic value of the consensus differentially expressed genes we identified comparing tumors to normal samples by intersecting the corresponding gene lists with those obtained by Cox proportional regression. Finally, in order to compare our results to previous prognostic analyses, we obtained data on enhancers position and prognostic potential from Chen et al. (2018), performed a liftOver to the hg38 genome assembly to match FC-R2 coordinates, and assessed the overlap between prognostic genes identified in the two studies.

## Data access

All data are available from http://marchionnilab.org/fcr2.html. Expression data can be directly accessed through https://jhubiostatistics.shinyapps.io/recount/ and the recount Bioconductor package (v1.9.5 or newer) at https://bioconductor.org/packages/recount as *RangedSummarizedExperiment* objects organized by the Sequence Read Archive (SRA) study ID. The data can be loaded using R-programming language and are ready to be analyzed using Bioconductor packages, or the data can be exported to other formats for use in another environment. All code used in this manuscript is available for reproducibility and transparency at GitHub (https://github.com/eddieimada/fcr2 and https://github.com/LieberInstitute/marchionni_projects). A compressed archive with all scripts used is also available as Supplemental Code.

## Competing interest statement

The authors declare no competing interests.

## Supplementary Material

Supplemental Material
