# Supplementary material for: Recounting the FANTOM CAGE-Associated Transcriptome
Source: Genome Res. 2020 Jul;30(7):1073–81. doi: 10.1101/gr.254656.119 (PMC7397872; doi:10.1101/gr.254656.119)
Supplement: Supplemental Material [file supp_30_7_1073__index.html]

Recounting the FANTOM CAGE-Associated Transcriptome — Supplemental Material 

# Recounting the FANTOM CAGE-Associated Transcriptome

## Supplemental Material

- SupplementaryCode.zip
- Supplemental\_Material.pdf
