# Supplementary material for: Recounting the FANTOM CAGE-Associated Transcriptome
Source: Genome Res. 2020 Jul;30(7):1073–81. doi: 10.1101/gr.254656.119 (PMC7397872; doi:10.1101/gr.254656.119)
Supplement: Supplemental Material [file supp_gr.254656.119_Supplemental_Material_.pdf]

Supplementary Information

Recounting the FANTOM Cage Associated Transcriptome

Eddie-Luidy Imada, Diego Fernando Sanchez, Leonardo Collado-Torres, Christopher Wilks, Tejasvi Matam, Wikum Dinalankara, Aleksey Stupnikov, Francisco Lobo-Pereira, Chi-Wai Yip, Kayoko Yasuzawa, Naoto Kondo, Masayoshi Itoh, Harukazu Suzuki, Takeya Kasukawa, Chung Chau Hon, Michiel de Hoon, Jay W Shin, Piero Carninci, FANTOM consortium, Andrew E Jaffe, Jeffrey T Leek, Alexander Favorov, Gloria R Franco, Ben Langmead, and Luigi Marchionni

Access to the data and code is available from http://marchionnilab.org/fcr2.html, expression data can be directly downloaded from https://jhubiostatistics.shinyapps.io/recount/ and the *recount* Bioconductor package (v1.9.5 or newer) at https://bioconductor.org/packages/recount.

Supplementary Tables

List of Tables

S1 Significant mRNAs across cancer types . . . 3

S2 Significant dp-lncRNAs across cancer types . . . 4

S3 Significant ip-lncRNAs across cancer types . . . 5

S4 Significant e-lncRNAs across cancer types . . . 5

S5 Percentage of differentially expressed genes in cancer that overlaps with GWAS SNPs associated with cancer . . . 6

S6 Andersson's enhancers in the FANTOM-CAT permissive set . . . 6

S7 Survival analysis for mRNAs using univariate Cox proportional regression . . . 7

S8 Survival analysis for dp-lncRNAs using univariate Cox proportional regression . . . 7

S9 Survival analysis for ip-lncRNAs using univariate Cox proportional regression . . . 8

S10 Survival analysis for e-lncRNAs using univariate Cox proportional regression . . . 8

S11 Prognostic differentially expressed mRNA genes across cancer types in univariate Cox proportional regression . . . 9

S12 Prognostic differentially expressed dp-lncRNA genes across cancer types in univariate Cox proportional regression . . . 10

S13 Prognostic differentially expressed ip-lncRNA genes across cancer types in univariate Cox proportional regression . . . 10

S14 Prognostic differentially expressed e-lncRNA genes across cancer types in univariate Cox proportional regression . . . 10

S15 Survival analysis for e-lncRNAs using multivariate Cox proportional regression . . . 11

S16 Survival analysis for dp-lncRNAs using multivariate Cox proportional regression . . . 11

S17 Survival analysis for ip-lncRNAs using multivariate Cox proportional regression . . . 12

S18 Survival analysis for mRNAs using multivariate Cox proportional regression . . . 12

S19 Prognostic differentially expressed mRNA genes across cancer types in multivariate Cox proportional regression. . . . 13

S20 Prognostic differentially expressed dp-lncRNA genes across cancer types in multivariate Cox proportional regression. . . . 14

S21 Prognostic differentially expressed ip-lncRNA genes across cancer types in multivariate Cox proportional regression. . . . 14

S22 Prognostic differentially expressed e-lncRNA genes across cancer types in multivariate Cox proportional regression. . . . 14

S23 List of new attributed HUGO Symbol. . . . 15

S24 List of features used in the multivariate Cox regression analysis. . . . 15

Table S1. Intersection of significant mRNAs across 13 cancer types (Global FDR < 0.000001)

geneID	geneSymbol	geneType	CAT_geneClass	CAT_DHS_type	Chromosome	geneStart	geneEnd	strand	Direction
ENSG00000065328	*MCM10*	protein_coding	coding_mRNA	DHS_promoter	chr10	13161579	13211916	+	Up-regulated in tumor
ENSG00000090889	*KIF4A*	protein_coding	coding_mRNA	DHS_promoter	chrX	70290040	70421061	+	Up-regulated in tumor
ENSG00000091651	*ORC6*	protein_coding	coding_mRNA	DHS_promoter	chr16	46689310	46702149	+	Up-regulated in tumor
ENSG00000092853	*CLSPN*	protein_coding	coding_mRNA	DHS_promoter	chr1	35719788	35769967	-	Up-regulated in tumor
ENSG00000093009	*CDC45*	protein_coding	coding_mRNA	DHS_promoter	chr22	19479466	19528387	+	Up-regulated in tumor
ENSG00000100162	*CENPM*	protein_coding	coding_mRNA	DHS_promoter	chr22	41769677	41947123	-	Up-regulated in tumor
ENSG00000102384	*CENPI*	protein_coding	coding_mRNA	DHS_promoter	chrX	101098204	101169831	+	Up-regulated in tumor
ENSG00000105011	*ASF1B*	protein_coding	coding_mRNA	DHS_promoter	chr19	14118278	14138467	-	Up-regulated in tumor
ENSG00000106268	*NUDT1*	protein_coding	coding_mRNA	DHS_promoter	chr7	2242226	2251354	+	Up-regulated in tumor
ENSG00000112984	*KIF20A*	protein_coding	coding_mRNA	DHS_promoter	chr5	138178724	138189480	+	Up-regulated in tumor
ENSG00000115163	*CENPA*	protein_coding	coding_mRNA	DHS_promoter	chr2	26764321	26803376	+	Up-regulated in tumor
ENSG00000117650	*NEK2*	protein_coding	coding_mRNA	DHS_promoter	chr1	211658373	211675621	-	Up-regulated in tumor
ENSG00000121152	*NCAPH*	protein_coding	coding_mRNA	DHS_promoter	chr2	96335801	96393940	+	Up-regulated in tumor
ENSG00000126787	*DLGAP5*	protein_coding	coding_mRNA	DHS_promoter	chr14	55148112	55191585	-	Up-regulated in tumor
ENSG00000138180	*CEP55*	protein_coding	coding_mRNA	DHS_promoter	chr10	93496639	93546919	+	Up-regulated in tumor
ENSG00000144554	*FANCD2*	protein_coding	coding_mRNA	DHS_promoter	chr3	10023970	10102460	+	Up-regulated in tumor
ENSG00000148773	*MKI67*	protein_coding	coding_mRNA	DHS_promoter	chr10	128092566	128126423	-	Up-regulated in tumor
ENSG00000156970	*BUB1B*	protein_coding	coding_mRNA	DHS_promoter	chr15	40161069	40223524	+	Up-regulated in tumor
ENSG00000157456	*CCNB2*	protein_coding	coding_mRNA	DHS_promoter	chr15	59097077	59161020	+	Up-regulated in tumor
ENSG00000162062	*TEDC2*	protein_coding	coding_mRNA	DHS_dyadic	chr16	2460109	2465114	+	Up-regulated in tumor
ENSG00000165304	*MELK*	protein_coding	coding_mRNA	DHS_promoter	chr9	36572085	36682572	+	Up-regulated in tumor
ENSG00000166508	*MCM7*	protein_coding	coding_mRNA	DHS_promoter	chr7	100085474	100105533	-	Up-regulated in tumor
ENSG00000167513	*CDT1*	protein_coding	coding_mRNA	DHS_promoter	chr16	88799649	88818226	+	Up-regulated in tumor
ENSG00000169679	*BUB1*	protein_coding	coding_mRNA	DHS_promoter	chr2	110630642	110678063	-	Up-regulated in tumor
ENSG00000186185	*KIF18B*	protein_coding	coding_mRNA	DHS_promoter	chr17	44923253	44947773	-	Up-regulated in tumor
ENSG00000189057	*FAM111B*	protein_coding	coding_mRNA	DHS_promoter	chr11	59063500	59131211	+	Up-regulated in tumor
ENSG00000076555	*ACACB*	protein_coding	coding_mRNA	DHS_enhancer	chr12	109116443	109270766	+	Down-regulated in tumor
ENSG00000106034	*CPED1*	protein_coding	coding_mRNA	DHS_promoter	chr7	120987841	121310413	+	Down-regulated in tumor
ENSG00000112425	*EPM2A*	protein_coding	coding_mRNA	DHS_promoter	chr6	145382951	145763619	-	Down-regulated in tumor
ENSG00000116678	*LEPR*	protein_coding	coding_mRNA	DHS_promoter	chr1	65420701	65643153	+	Down-regulated in tumor
ENSG00000130988	*RGN*	protein_coding	coding_mRNA	DHS_promoter	chrX	47078366	47104867	+	Down-regulated in tumor
ENSG00000133800	*LYVE1*	protein_coding	coding_mRNA	DHS_enhancer	chr11	10307853	10611707	-	Down-regulated in tumor
ENSG00000136842	*TMOD1*	protein_coding	coding_mRNA	DHS_promoter	chr9	97501142	97628196	+	Down-regulated in tumor
ENSG00000138356	*AOX1*	protein_coding	coding_mRNA	DHS_promoter	chr2	200586053	200677064	+	Down-regulated in tumor
ENSG00000151623	*NR3C2*	protein_coding	coding_mRNA	DHS_promoter	chr4	148077496	148445575	-	Down-regulated in tumor
ENSG00000154330	*PGM5*	protein_coding	coding_mRNA	DHS_promoter	chr9	68327407	68539746	+	Down-regulated in tumor

(Continuing from previous page)

geneID	geneSymbol	geneType	CAT_geneClass	CAT_DHS_type	Chromosome	geneStart	geneEnd	strand	Direction
ENSG00000168546	*GFRA2*	protein_coding	coding_mRNA	DHS_promoter	chr8	21365101	21812338	-	Down-regulated in tumor
ENSG00000170271	*FAXDC2*	protein_coding	coding_mRNA	DHS_promoter	chr5	154810519	154859509	-	Down-regulated in tumor
ENSG00000185432	*METTL7A*	protein_coding	coding_mRNA	DHS_promoter	chr12	50922629	50935894	+	Down-regulated in tumor
ENSG00000196616	*ADH1B*	protein_coding	coding_mRNA	DHS_enhancer	chr4	99286865	99321401	-	Down-regulated in tumor
ENSG00000198300	*PEG3*	protein_coding	coding_mRNA	DHS_promoter	chr19	56801328	56840726	-	Down-regulated in tumor

Table S2. Intersection of significant divergent promoters across 13 cancer types (Global FDR < 0.1)

geneID	geneSymbol	geneType	CAT_geneClass	CAT_DHS_type	Chromosome	geneStart	geneEnd	strand	Direction
CATG00000017193	*SUGT1-DT*	__na	lncRNA_divergent	DHS_promoter	chr13	52649609	52652307	-	Up-regulated in tumor
CATG00000020461	*CATG00000020461.1*	__na	lncRNA_divergent	DHS_promoter	chr14	22831988	22836822	-	Up-regulated in tumor
CATG00000054098	*CATG00000054098.1*	__na	lncRNA_divergent	DHS_promoter	chr20	3043468	3048131	-	Up-regulated in tumor
CATG00000087995	*CATG00000087995.1*	__na	lncRNA_divergent	DHS_promoter	chr6	30639697	30647759	-	Up-regulated in tumor
CATG00000101363	*CATG00000101363.1*	__na	lncRNA_divergent	DHS_promoter	chr8	144792564	144796174	+	Up-regulated in tumor
ENSG00000228839	*PIK3IP1-DT*	antisense	lncRNA_divergent	DHS_promoter	chr22	31290906	31357952	+	Up-regulated in tumor
ENSG00000235989	*MORC2-AS1*	antisense	lncRNA_divergent	DHS_promoter	chr22	30922325	30932449	+	Up-regulated in tumor
ENSG00000247373	*TMED2-DT*	lincRNA	lncRNA_divergent	DHS_promoter	chr12	123575002	123584820	-	Up-regulated in tumor
ENSG00000255717	*SNHG1*	processed_transcript	lncRNA_divergent	DHS_promoter	chr11	62851874	62855885	-	Up-regulated in tumor
ENSG00000257605	*MYG1-AS1*	antisense	lncRNA_divergent	DHS_promoter	chr12	53298394	53300360	-	Up-regulated in tumor
ENSG00000258384	*RCCD1-AS1*	antisense	lncRNA_divergent	DHS_promoter	chr15	90950782	90955229	-	Up-regulated in tumor
ENSG00000260442	*ATP2A1-AS1*	antisense	lncRNA_divergent	DHS_promoter	chr16	28868384	28879920	-	Up-regulated in tumor
ENSG00000263412	*NFE2L1-DT*	processed_transcript	lncRNA_divergent	DHS_promoter	chr17	48038796	48048670	-	Up-regulated in tumor
ENSG00000270195	*SLBP-DT*	lincRNA	lncRNA_divergent	DHS_promoter	chr4	1712410	1715967	+	Up-regulated in tumor
ENSG00000272455	*MRPL20-DT*	lincRNA	lncRNA_divergent	DHS_promoter	chr1	1407377	1410854	+	Up-regulated in tumor
CATG00000105517	*KLF9-DT*	__na	lncRNA_divergent	DHS_promoter	chr9	70413086	70609298	+	Down-regulated in tumor
ENSG00000175611	*LINC00476*	processed_transcript	lncRNA_divergent	DHS_promoter	chr9	95759231	95875979	-	Down-regulated in tumor
ENSG00000225793	*TMEM30A-DT*	lincRNA	lncRNA_divergent	DHS_promoter	chr6	75284598	75305766	+	Down-regulated in tumor
ENSG00000226237	*GAS1RR*	lincRNA	lncRNA_divergent	DHS_promoter	chr9	86946617	87014413	+	Down-regulated in tumor
ENSG00000232160	*RAP2C-AS1*	antisense	lncRNA_divergent	DHS_promoter	chrX	132217007	132435459	+	Down-regulated in tumor
ENSG00000234492	*RPL34-DT*	lincRNA	lncRNA_divergent	DHS_promoter	chr4	108538190	108620395	-	Down-regulated in tumor
ENSG00000235652	*FBXO30-DT*	antisense	lncRNA_divergent	DHS_promoter	chr6	145814895	145886585	+	Down-regulated in tumor
ENSG00000245293	*CYP2U1-AS1*	antisense	lncRNA_divergent	DHS_promoter	chr4	107862676	107989692	-	Down-regulated in tumor
ENSG00000248866	*USP46-DT*	lincRNA	lncRNA_divergent	DHS_promoter	chr4	52656631	52669444	+	Down-regulated in tumor
ENSG00000248980	*SPCS3-AS1*	antisense	lncRNA_divergent	DHS_promoter	chr4	176303507	176322213	-	Down-regulated in tumor
ENSG00000267414	*SETBP1-DT*	lincRNA	lncRNA_divergent	DHS_promoter	chr18	44664515	44680693	-	Down-regulated in tumor

Continued on next page

(Continuing from previous page)

geneID	geneSymbol	geneType	CAT_geneClass	CAT_DHS_type	Chromosome	geneStart	geneEnd	strand	Direction
ENSG00000271849	*MAN2A1-DT*	lincRNA	lncRNA_divergent	DHS_promoter	chr5	109687802	109689251	-	Down-regulated in tumor
ENSG00000272686	*WASL-DT*	antisense	lncRNA_divergent	DHS_promoter	chr7	123748542	123756392	+	Down-regulated in tumor

Table S3. Intersection of significant intergenic promoters across 13 cancer types (Global FDR < 0.1)

geneID	geneSymbol	geneType	CAT_geneClass	CAT_DHS_type	Chromosome	geneStart	geneEnd	strand	Direction
ENSG00000196756	*SNHG17*	processed_transcript	lncRNA_intergenic	DHS_promoter	chr20	38404766	38435328	-	Up-regulated in tumor
ENSG00000260924	*LINC01311*	antisense	lncRNA_intergenic	DHS_promoter	chr22	19171395	19175403	+	Up-regulated in tumor
ENSG00000215386	*MIR99AHG*	lincRNA	lncRNA_intergenic	DHS_promoter	chr21	15910380	16646424	+	Down-regulated in tumor
ENSG00000263753	*LINC00667*	lincRNA	lncRNA_intergenic	DHS_promoter	chr18	5237844	5251731	+	Down-regulated in tumor

Table S4. Intersection of significant enhancers across 13 cancer types (Global FDR < 0.1)

geneID	geneSymbol	geneType	CAT_geneClass	CAT_DHS_type	Chromosome	geneStart	geneEnd	strand	Direction
CATG00000107122	*CATG00000107122.1*	__na	lncRNA_antisense	DHS_enhancer	chr9	128118082	128124306	+	Up-regulated in tumor
ENSG00000231246	*LINC02884*	lincRNA	lncRNA_intergenic	DHS_enhancer	chr1	112176973	112360607	-	Down-regulated in tumor
ENSG00000255958	*GABARAPL1-AS1*	antisense	lncRNA_antisense	DHS_enhancer	chr12	10214161	10220555	-	Down-regulated in tumor

Table S5. Percentage of differentially expressed genes in cancer that overlaps with GWAS SNPs associated with cancer. In the table are reported the percentage of significant DEG ($FDR \leq 0.01$) between tumor and normal samples across the 13 cancer types analyzed for each gene class considered (mRNA, ip-lncRNA, dp-lncRNA, and e-lncRNA) that overlaps with GWAS SNPs associated with cancer. The list of GWAS SNPs was obtained from the GWAS Catalog (Buniello et al. 2018).

Cancer Type	d-lncRNA	e-lncRNA	i-lnRNA	mRNA
Bile	5.85%	6.94%	12.21%	9.10%
Bladder	5.28%	6.92%	10.88%	10.15%
Breast	5.22%	6.46%	9.87%	9.30%
Colorectal	5.64%	7.04%	9.66%	9.02%
Esophagus	7.14%	8.03%	10.53%	9.79%
Head and Neck	6.64%	9.85%	9.09%	9.59%
Kidney	4.50%	6.27%	9.93%	9.14%
Liver	5.67%	6.49%	12.38%	9.39%
Lung	4.83%	5.67%	8.79%	9.03%
Prostate	4.93%	5.98%	10.53%	9.06%
Stomach	5.64%	5.46%	9.89%	8.83%
Thyroid	4.50%	5.71%	9.55%	9.30%
Uterus	4.36%	5.53%	9.43%	9.48%

Table S6. Remapping of Andersson's enhancers list to the FANTOM-CAT permissive set. Since originally published, many of enhancers contained in the Andersson's list (Andersson et al. 2014), were reassigned or removed during the assembly of the *FANTOM-CAT* based on further evidence from the additional transcriptomic datasets used in the meta-assembly, as well as due to the information obtained from orthogonal genomic and epigenomic information, such as DNase I hypersensitivity and other epigenomic marks, as obtained from the Roadmap Epigenomics Project. Based on the new gene models in *FANTOM-CAT*, we verified the overlapping between the original enhancers list and then summarized the results according to current RNA classes in the *FANTOM-CAT*. The counts for the original enhancers that overlap with exons in the FANTOM-CAT gene models are shown in the "Exonic" column on the left. The counts for the enhancers that did not map to any exon, but that were still within the gene boundaries are shown in the "Intronic" column on the right.

	Exonic	Intronic
d-lncRNA	1628	501
e-lncRNA	7882	1647
i-lncRNA	997	273
mRNA	10194	7197
other-RNA	6893	2100
pseudogene	1073	204
senseOverlap-RNA	2289	150
small-RNA	635	65
Total	31591	12137

Table S7. Survival analysis using univariate Cox proportional regression showing the number of mRNAs with prognostic value accross the 13 cancer types. *Non-significant* column indicates the number of genes with FDR greater than 0.05. *Cases* represents the number patients at the beginning of follow-up for each tumor type. *Events* is the number of death cases during follow up. *Median time* is given in days.

Tumor type	Non-significant	FDR $\leq$ 0.05	Cases	Events	Median time
Kidney	10126	11973	881	227	N.A.
Uterus	14929	7169	596	125	3365
Liver	19505	2587	365	130	1694
Bladder	21315	783	407	178	1008
Head and Neck	21793	305	501	217	1671
Stomach	21998	101	392	158	940
Lung	22026	73	998	395	1531
Breast	22091	8	1080	151	3941
Thyroid	22090	5	504	16	N.A.
Colorectal	22095	1	602	128	2532
Prostate	22095	1	496	10	N.A.
Bile	21952	0	36	18	1220
Esophagus	22099	0	184	77	784

Table S8. Survival analysis using univariate Cox proportional regression showing the number of dp-lncRNAs with prognostic value accross the 13 cancer types. *Non-significant* column indicates the number of genes with FDR greater than 0.05. *Cases* represents the number patients at the beginning of follow-up for each tumor type. *Events* is the number of death cases during follow up. *Median time* is given in days.

Tumor type	Non-significant	FDR $\leq$ 0.05	Cases	Events	Median time
Kidney	3309	2895	881	227	N.A.
Uterus	5190	1013	596	125	3365
Bladder	5884	319	407	178	1008
Liver	6093	101	365	130	1694
Stomach	6194	10	392	158	940
Colorectal	6197	5	602	128	2532
HeadNeck	6199	5	501	217	1671
Breast	6200	3	1080	151	3941
Bile	6078	0	36	18	1220
Esophagus	6204	0	184	77	784
Lung	6204	0	998	395	1531
Prostate	6203	0	496	10	N.A.
Thyroid	6201	0	504	16	N.A.

Table S9. Survival analysis using univariate Cox proportional regression showing the number of ip-lncRNAs with prognostic value accross the 13 cancer types. *Non-significant* column indicates the number of genes with FDR greater than 0.05. *Cases* represents the number patients at the beginning of follow-up for each tumor type. *Events* is the number of death cases during follow up. *Median time* is given in days.

Tumor type	Non-significant	FDR $\leq$ 0.05	Cases	Events	Median time
Kidney	982	966	881	227	N.A.
Uterus	1519	428	596	125	3365
Liver	1884	58	365	130	1694
Bladder	1936	12	407	178	1008
Head and Neck	1942	5	501	217	1671
Colorectal	1938	4	602	128	2532
Stomach	1944	4	392	158	940
Prostate	1945	2	496	10	N.A.
Lung	1946	1	998	395	1531
Thyroid	1945	1	504	16	N.A.
Bile	1865	0	36	18	1220
Breast	1948	0	1080	151	3941
Esophagus	1947	0	184	77	784

Table S10. Survival analysis using univariate Cox proportional regression showing the number of e-lncRNAs with prognostic value accross the 13 cancer types. *Non-significant* column indicates the number of genes with FDR greater than 0.05. *Cases* represents the number patients at the beginning of follow-up for each tumor type. *Events* is the number of death cases during follow up. *Median time* is given in days.

Tumor type	Non-significant	FDR $\leq$ 0.05	Cases	Events	Median time
Kidney	500	1200	881	227	N.A.
Uterus	1871	551	596	125	3365
Bladder	1066	105	407	178	1008
Head and Neck	1122	18	501	217	1671
Liver	976	15	365	130	1694
Lung	1535	9	998	395	1531
Breast	2039	7	1080	151	3941
Bile	1237	0	36	18	1220
Colorectal	1830	0	602	128	2532
Esophagus	2482	0	184	77	784
Prostate	2896	0	496	10	N.A.
Stomach	2663	0	392	158	940
Thyroid	2623	0	504	16	N.A.

Table S11. Differentially expressed mRNA genes with prognostic value across cancer types using univariate Cox proportional regression. *N.P.* refers that the given gene is non-prognostic on this cancer type

	Bladder	Breast	Colorectal	Head and Neck	Kidney	Liver	Lung	Prostate	Stomach	Thyroid	Uterus
MCM10	N.P.	N.P.	N.P.	N.P.	1.6 (1.43-1.79)	1.3 (1.17-1.46)	N.P.	N.P.	N.P.	N.P.	1.36 (1.17-1.58)
KIF4A	N.P.	N.P.	N.P.	N.P.	1.7 (1.54-1.89)	1.23 (1.11-1.36)	N.P.	N.P.	N.P.	N.P.	1.37 (1.15-1.63)
ORC6	N.P.	N.P.	N.P.	N.P.	1.7 (1.49-1.93)	1.3 (1.15-1.48)	N.P.	N.P.	N.P.	N.P.	1.35 (1.1-1.66)
CLSPN	N.P.	N.P.	N.P.	N.P.	1.63 (1.44-1.85)	1.31 (1.17-1.46)	N.P.	N.P.	N.P.	N.P.	1.29 (1.09-1.52)
CDC45	N.P.	N.P.	N.P.	N.P.	1.71 (1.51-1.94)	1.31 (1.16-1.49)	N.P.	N.P.	N.P.	N.P.	1.24 (1.04-1.48)
CENPM	N.P.	N.P.	N.P.	N.P.	1.42 (1.26-1.6)	1.25 (1.12-1.4)	N.P.	N.P.	N.P.	N.P.	N.P.
CENPI	N.P.	N.P.	N.P.	N.P.	1.87 (1.67-2.1)	1.3 (1.15-1.47)	N.P.	N.P.	N.P.	N.P.	1.41 (1.16-1.72)
ASF1B	N.P.	N.P.	N.P.	N.P.	1.69 (1.48-1.92)	1.23 (1.09-1.38)	N.P.	N.P.	N.P.	N.P.	N.P.
NUDT1	N.P.	N.P.	N.P.	N.P.	1.49 (1.27-1.75)	N.P.	N.P.	N.P.	N.P.	N.P.	1.3 (1.09-1.54)
KIF20A	N.P.	N.P.	N.P.	N.P.	1.78 (1.62-1.95)	1.38 (1.22-1.56)	N.P.	N.P.	N.P.	N.P.	1.41 (1.17-1.7)
CENPA	N.P.	N.P.	N.P.	N.P.	1.79 (1.63-1.96)	1.32 (1.19-1.48)	N.P.	N.P.	N.P.	N.P.	1.54 (1.27-1.86)
NEK2	N.P.	N.P.	N.P.	N.P.	1.67 (1.52-1.84)	1.21 (1.09-1.34)	N.P.	N.P.	N.P.	N.P.	N.P.
NCAPH	N.P.	N.P.	N.P.	N.P.	1.85 (1.65-2.08)	1.26 (1.12-1.42)	N.P.	N.P.	N.P.	N.P.	1.45 (1.2-1.76)
DLGAP5	N.P.	N.P.	N.P.	N.P.	1.59 (1.44-1.75)	1.29 (1.16-1.44)	N.P.	N.P.	N.P.	N.P.	1.34 (1.15-1.56)
CEP55	N.P.	N.P.	N.P.	N.P.	1.8 (1.63-1.99)	1.32 (1.18-1.48)	N.P.	N.P.	N.P.	N.P.	N.P.
FANCD2	N.P.	N.P.	N.P.	N.P.	1.75 (1.48-2.08)	1.27 (1.11-1.45)	N.P.	N.P.	N.P.	N.P.	1.32 (1.07-1.63)
MKI67	N.P.	N.P.	N.P.	N.P.	1.63 (1.47-1.81)	1.3 (1.16-1.46)	N.P.	N.P.	N.P.	N.P.	1.26 (1.05-1.51)
BUB1B	N.P.	N.P.	N.P.	N.P.	1.73 (1.55-1.93)	1.23 (1.11-1.37)	N.P.	N.P.	N.P.	N.P.	1.33 (1.12-1.58)
CCNB2	N.P.	N.P.	N.P.	N.P.	1.96 (1.75-2.2)	1.21 (1.07-1.37)	N.P.	N.P.	N.P.	N.P.	1.3 (1.08-1.57)
TEDC2	N.P.	N.P.	N.P.	N.P.	1.19 (1.08-1.32)	1.28 (1.13-1.45)	N.P.	N.P.	N.P.	N.P.	1.38 (1.13-1.68)
MELK	N.P.	N.P.	N.P.	N.P.	1.79 (1.59-2.02)	1.29 (1.15-1.45)	N.P.	N.P.	N.P.	N.P.	1.26 (1.06-1.49)
MCM7	N.P.	N.P.	N.P.	N.P.	1.31 (1.07-1.6)	1.33 (1.13-1.56)	N.P.	N.P.	N.P.	N.P.	1.45 (1.18-1.77)
CDT1	N.P.	N.P.	N.P.	N.P.	1.51 (1.28-1.77)	1.28 (1.13-1.44)	N.P.	N.P.	N.P.	N.P.	N.P.
BUB1	N.P.	N.P.	N.P.	N.P.	1.72 (1.55-1.91)	1.32 (1.17-1.49)	N.P.	N.P.	N.P.	N.P.	1.55 (1.28-1.87)
KIF18B	N.P.	N.P.	N.P.	N.P.	1.75 (1.6-1.92)	1.24 (1.12-1.37)	N.P.	N.P.	N.P.	N.P.	1.43 (1.19-1.7)
FAM111B	N.P.	N.P.	N.P.	N.P.	1.37 (1.22-1.54)	1.2 (1.06-1.35)	N.P.	N.P.	N.P.	N.P.	N.P.
ACACB	N.P.	N.P.	N.P.	N.P.	0.79 (0.71-0.88)	N.P.	N.P.	N.P.	N.P.	N.P.	1.45 (1.24-1.7)
CPED1	1.16 (1.06-1.28)	N.P.	N.P.	N.P.	1.1 (1.02-1.18)	N.P.	N.P.	N.P.	N.P.	N.P.	N.P.
EPM2A	N.P.	N.P.	N.P.	N.P.	0.6 (0.49-0.72)	N.P.	N.P.	N.P.	N.P.	N.P.	1.56 (1.26-1.94)
RGN	N.P.	N.P.	N.P.	N.P.	0.74 (0.68-0.8)	0.86 (0.78-0.95)	N.P.	N.P.	N.P.	N.P.	1.15 (1.04-1.26)
LYVE1	N.P.	N.P.	N.P.	N.P.	1.11 (1.04-1.2)	N.P.	N.P.	N.P.	N.P.	N.P.	1.38 (1.2-1.58)
AOX1	N.P.	N.P.	N.P.	N.P.	N.P.	N.P.	N.P.	N.P.	N.P.	N.P.	1.17 (1.07-1.28)
NR3C2	N.P.	N.P.	N.P.	N.P.	0.79 (0.73-0.84)	N.P.	N.P.	N.P.	N.P.	N.P.	N.P.
PGM5	N.P.	N.P.	N.P.	N.P.	0.82 (0.75-0.89)	N.P.	N.P.	N.P.	N.P.	N.P.	N.P.
GFRA2	N.P.	N.P.	N.P.	N.P.	1.21 (1.12-1.31)	N.P.	N.P.	N.P.	N.P.	N.P.	1.19 (1.09-1.3)
FAXDC2	N.P.	N.P.	N.P.	N.P.	0.79 (0.7-0.88)	N.P.	N.P.	N.P.	N.P.	N.P.	N.P.
METTL7A	N.P.	N.P.	N.P.	N.P.	0.72 (0.66-0.78)	N.P.	N.P.	N.P.	N.P.	N.P.	N.P.
ADH1B	N.P.	N.P.	N.P.	N.P.	N.P.	0.9 (0.85-0.96)	N.P.	N.P.	N.P.	N.P.	N.P.
PEG3	N.P.	N.P.	N.P.	N.P.	0.86 (0.79-0.95)	N.P.	N.P.	N.P.	N.P.	N.P.	N.P.

Table S12. Differentially expressed dp-lncRNA genes with prognostic value across cancer types using univariate Cox proportional regression. *N.P.* refers that the given gene is non-prognostic on this cancer type

	Bladder	Breast	Colorectal	Kidney	Liver	Lung	Prostate	Stomach	Thyroid	Uterus
SUGT1-DT	0.78 (0.67-0.9)	N.P.	N.P.	1.13 (1.02-1.25)	N.P.	N.P.	N.P.	N.P.	N.P.	N.P.
CATG00000020461.1	N.P.	N.P.	N.P.	1.26 (1.14-1.39)	N.P.	N.P.	N.P.	N.P.	N.P.	N.P.
CATG00000054098.1	0.79 (0.68-0.91)	N.P.	N.P.	1.22 (1.1-1.36)	N.P.	N.P.	N.P.	N.P.	N.P.	N.P.
CATG00000087995.1	0.78 (0.69-0.88)	N.P.	N.P.	1.13 (1.02-1.25)	N.P.	N.P.	N.P.	N.P.	N.P.	N.P.
CATG00000101363.1	N.P.	N.P.	N.P.	N.P.	N.P.	N.P.	N.P.	N.P.	N.P.	1.36 (1.18-1.56)
PIK3IP1-DT	N.P.	N.P.	N.P.	N.P.	N.P.	N.P.	N.P.	N.P.	N.P.	0.8 (0.68-0.94)
MORC2-AS1	0.76 (0.66-0.88)	N.P.	1.42 (1.2-1.68)	1.37 (1.25-1.52)	N.P.	N.P.	N.P.	N.P.	N.P.	N.P.
TMED2-DT	N.P.	N.P.	N.P.	1.26 (1.14-1.4)	N.P.	N.P.	N.P.	N.P.	N.P.	N.P.
SNHG1	N.P.	N.P.	N.P.	1.45 (1.24-1.71)	N.P.	N.P.	N.P.	N.P.	N.P.	1.44 (1.16-1.78)
MYG1-AS1	N.P.	N.P.	N.P.	1.68 (1.48-1.9)	1.33 (1.13-1.57)	N.P.	N.P.	N.P.	N.P.	1.42 (1.15-1.75)
RCCD1-AS1	N.P.	N.P.	N.P.	1.47 (1.31-1.65)	N.P.	N.P.	N.P.	N.P.	N.P.	N.P.
ATP2A1-AS1	N.P.	N.P.	N.P.	1.18 (1.06-1.31)	N.P.	N.P.	N.P.	N.P.	N.P.	N.P.
NFE2L1-DT	0.77 (0.66-0.9)	N.P.	N.P.	1.24 (1.11-1.38)	N.P.	N.P.	N.P.	N.P.	N.P.	N.P.
SLBP-DT	0.76 (0.66-0.88)	N.P.	N.P.	1.28 (1.16-1.4)	N.P.	N.P.	N.P.	N.P.	N.P.	N.P.
MRPL20-DT	N.P.	N.P.	N.P.	1.48 (1.29-1.69)	N.P.	N.P.	N.P.	N.P.	N.P.	1.55 (1.32-1.82)
KLF9-DT	N.P.	N.P.	N.P.	N.P.	N.P.	N.P.	N.P.	N.P.	N.P.	1.36 (1.11-1.66)
GAS1RR	N.P.	N.P.	N.P.	N.P.	N.P.	N.P.	N.P.	N.P.	N.P.	1.26 (1.14-1.39)
RAP2C-AS1	N.P.	N.P.	N.P.	0.68 (0.56-0.81)	N.P.	N.P.	N.P.	N.P.	N.P.	N.P.
RPL34-DT	N.P.	N.P.	N.P.	0.7 (0.59-0.82)	N.P.	N.P.	N.P.	N.P.	N.P.	N.P.
FBXO30-DT	N.P.	N.P.	N.P.	0.86 (0.77-0.96)	N.P.	N.P.	N.P.	N.P.	N.P.	N.P.
SPCS3-AS1	N.P.	N.P.	N.P.	0.8 (0.68-0.95)	N.P.	N.P.	N.P.	N.P.	N.P.	N.P.
SETBP1-DT	1.22 (1.09-1.36)	N.P.	N.P.	0.78 (0.67-0.91)	N.P.	N.P.	N.P.	N.P.	N.P.	1.26 (1.08-1.47)
MAN2A1-DT	N.P.	N.P.	N.P.	0.8 (0.7-0.9)	N.P.	N.P.	N.P.	N.P.	N.P.	N.P.
WASL-DT	N.P.	N.P.	N.P.	0.79 (0.66-0.94)	N.P.	N.P.	N.P.	N.P.	N.P.	N.P.

Table S13. Differentially expressed ip-lncRNA genes with prognostic value across cancer types using univariate Cox proportional regression. *N.P.* refers that the given gene is non-prognostic on this cancer type

	Bladder	Breast	Colorectal	Head and Neck	Kidney	Liver	Lung	Prostate	Stomach	Thyroid	Uterus
SNHG17	N.P.	N.P.	N.P.	N.P.	1.7 (1.45-1.99)	N.P.	N.P.	N.P.	N.P.	N.P.	1.53 (1.25-1.86)
LINC01311	N.P.	N.P.	N.P.	N.P.	1.15 (1.03-1.29)	N.P.	N.P.	N.P.	N.P.	N.P.	N.P.
MIR99AHG	N.P.	N.P.	N.P.	0.85 (0.78-0.92)	N.P.	N.P.	N.P.	N.P.	N.P.	N.P.	1.22 (1.09-1.35)
LINC00667	N.P.	N.P.	N.P.	N.P.	N.P.	N.P.	N.P.	N.P.	N.P.	N.P.	1.25 (1.12-1.39)

Table S14. Differentially expressed e-lncRNA genes with prognostic value across cancer types using univariate Cox proportional regression. *N.P.* refers that the given gene is non-prognostic on this cancer type

	Bladder	Breast	Colorectal	Head and Neck	Kidney	Liver	Lung	Prostate	Stomach	Thyroid	Uterus
CATG00000107122.1	0.79 (0.67-0.92)	N.P.	N.P.	N.P.	1.13 (1.03-1.24)	N.P.	N.P.	N.P.	N.P.	N.P.	N.P.
LINC02884	N.P.	N.P.	N.P.	N.P.	1.14 (1.06-1.22)	N.P.	N.P.	N.P.	N.P.	N.P.	N.P.

Table S15. Survival analysis using multivariate Cox proportional regression showing the number of e-lncRNAs with prognostic value accross the 13 cancer types. *Non-significant* column indicates the number of genes with FDR greater than 0.05. *Cases* represents the number patients at the beginning of follow-up for each tumor type. *Events* is the number of death cases during follow up. *Median time* is given in days.

Tumor type	Non-significant	FDR $\leq$ 0.05	Cases	Events	Median time
Kidney	776	1175	881	227	N.A.
Uterus	2083	428	596	125	3365
Bladder	1221	79	407	178	1008
Liver	1123	6	365	130	1694
Head and Neck	1250	3	501	217	1671
Breast	2489	2	1080	151	3941
Colorectal	1919	1	602	128	2532
Bile	1286	0	36	18	1220
Esophagus	2621	0	184	77	784
Lung	1770	0	998	395	1531
Prostate	3191	0	496	10	N.A.
Stomach	2759	0	392	158	940
Thyroid	2862	0	504	16	N.A.

Table S16. Survival analysis using multivariate Cox proportional regression showing the number of dp-lncRNAs with prognostic value accross the 13 cancer types. *Non-significant* column indicates the number of genes with FDR greater than 0.05. *Cases* represents the number patients at the beginning of follow-up for each tumor type. *Events* is the number of death cases during follow up. *Median time* is given in days.

Tumor type	Non-significant	FDR $\leq$ 0.05	Cases	Events	Median time
Kidney	3168	2595	881	227	N.A.
Uterus	5326	656	596	125	3365
Bladder	5642	53	407	178	1008
Stomach	5995	16	392	158	940
Liver	5827	12	365	130	1694
Breast	6011	3	1080	151	3941
Colorectal	5939	1	602	128	2532
Lung	5863	1	998	395	1531
Bile	5736	0	36	18	1220
Esophagus	5928	0	184	77	784
HeadNeck	5954	0	501	217	1671
Prostate	5843	0	496	10	N.A.
Thyroid	6177	0	504	16	N.A.

Table S17. Survival analysis using multivariate Cox proportional regression showing the number of ip-lncRNAs with prognostic value accross the 13 cancer types. *Non-significant* column indicates the number of genes with FDR greater than 0.05. *Cases* represents the number patients at the beginning of follow-up for each tumor type. *Events* is the number of death cases during follow up. *Median time* is given in days.

Tumor type	Non-significant	FDR ≤ 0.05	Cases	Events	Median time
Kidney	1063	759	881	227	N.A.
Uterus	1591	295	596	125	3365
Stomach	1895	17	392	158	940
Liver	1796	11	365	130	1694
Bladder	1798	4	407	178	1008
Lung	1855	2	998	395	1531
Prostate	1832	1	496	10	N.A.
Bile	1778	0	36	18	1220
Breast	1896	0	1080	151	3941
Colorectal	1863	0	602	128	2532
Esophagus	1873	0	184	77	784
Head and Neck	1856	0	501	217	1671
Thyroid	1937	0	504	16	N.A.

Table S18. Survival analysis using multivariate Cox proportional regression showing the number of mRNAs with prognostic value accross the 13 cancer types. *Non-significant* column indicates the number of genes with FDR greater than 0.05. *Cases* represents the number patients at the beginning of follow-up for each tumor type. *Events* is the number of death cases during follow up. *Median time* is given in days.

Tumor type	Non-significant	FDR ≤ 0.05	Cases	Events	Median time
Kidney	12626	8161	881	227	N.A.
Uterus	15850	5252	596	125	3365
Bladder	19267	262	407	178	1008
Liver	18234	219	365	130	1694
Stomach	20961	117	392	158	940
Lung	20776	15	998	395	1531
Bile	21131	0	36	18	1220
Breast	21004	0	1080	151	3941
Colorectal	21262	0	602	128	2532
Esophagus	20927	0	184	77	784
Head and Neck	21427	0	501	217	1671
Prostate	20697	0	496	10	N.A.
Thyroid	22036	0	504	16	N.A.

Table S19. Differentially expressed mRNA genes with prognostic value across cancer types using multivariate Cox proportional regression. *N.P.* refers that the given gene is non-prognostic on this cancer type

	Bladder	Breast	Colorectal	Head and Neck	Kidney	Liver	Lung	Prostate	Stomach	Thyroid	Uterus
MCM10	N.P.	N.P.	N.P.	N.P.	1.4 (1.22-1.6)	N.P.	N.P.	N.P.	N.P.	N.P.	1.32 (1.13-1.54)
KIF4A	N.P.	N.P.	N.P.	N.P.	1.53 (1.35-1.74)	N.P.	N.P.	N.P.	N.P.	N.P.	1.32 (1.1-1.58)
ORC6	N.P.	N.P.	N.P.	N.P.	1.73 (1.5-1.99)	N.P.	N.P.	N.P.	N.P.	N.P.	1.38 (1.1-1.72)
CLSPN	N.P.	N.P.	N.P.	N.P.	1.43 (1.23-1.67)	N.P.	N.P.	N.P.	N.P.	N.P.	N.P.
CDC45	N.P.	N.P.	N.P.	N.P.	1.43 (1.24-1.66)	N.P.	N.P.	N.P.	N.P.	N.P.	N.P.
CENPM	N.P.	N.P.	N.P.	N.P.	1.39 (1.2-1.62)	N.P.	N.P.	N.P.	N.P.	N.P.	N.P.
CENPI	N.P.	N.P.	N.P.	N.P.	1.68 (1.46-1.93)	N.P.	N.P.	N.P.	N.P.	N.P.	1.39 (1.13-1.72)
ASF1B	N.P.	N.P.	N.P.	N.P.	1.35 (1.16-1.58)	N.P.	N.P.	N.P.	N.P.	N.P.	N.P.
NUDT1	N.P.	N.P.	N.P.	N.P.	1.66 (1.37-2.01)	N.P.	N.P.	N.P.	N.P.	N.P.	1.32 (1.1-1.58)
KIF20A	N.P.	N.P.	N.P.	N.P.	1.46 (1.31-1.63)	N.P.	N.P.	N.P.	N.P.	N.P.	1.34 (1.11-1.63)
CENPA	N.P.	N.P.	N.P.	N.P.	1.57 (1.4-1.76)	N.P.	N.P.	N.P.	N.P.	N.P.	1.5 (1.23-1.83)
NEK2	N.P.	N.P.	N.P.	N.P.	1.48 (1.32-1.67)	N.P.	N.P.	N.P.	N.P.	N.P.	N.P.
NCAPH	N.P.	N.P.	N.P.	N.P.	1.55 (1.35-1.78)	N.P.	N.P.	N.P.	N.P.	N.P.	1.41 (1.15-1.72)
DLGAP5	N.P.	N.P.	N.P.	N.P.	1.4 (1.25-1.58)	N.P.	N.P.	N.P.	N.P.	N.P.	1.33 (1.13-1.56)
CEP55	N.P.	N.P.	N.P.	N.P.	1.53 (1.36-1.74)	1.29 (1.14-1.46)	N.P.	N.P.	N.P.	N.P.	N.P.
FANCD2	N.P.	N.P.	N.P.	N.P.	1.68 (1.39-2.04)	N.P.	N.P.	N.P.	N.P.	N.P.	N.P.
MKI67	N.P.	N.P.	N.P.	N.P.	1.39 (1.22-1.57)	N.P.	N.P.	N.P.	N.P.	N.P.	N.P.
BUB1B	N.P.	N.P.	N.P.	N.P.	1.55 (1.35-1.77)	N.P.	N.P.	N.P.	N.P.	N.P.	1.28 (1.07-1.53)
CCNB2	N.P.	N.P.	N.P.	N.P.	1.9 (1.64-2.19)	N.P.	N.P.	N.P.	N.P.	N.P.	N.P.
TEDC2	N.P.	N.P.	N.P.	N.P.	1.47 (1.28-1.7)	N.P.	N.P.	N.P.	N.P.	N.P.	N.P.
MELK	N.P.	N.P.	N.P.	N.P.	1.51 (1.31-1.74)	N.P.	N.P.	N.P.	N.P.	N.P.	N.P.
MCM7	N.P.	N.P.	N.P.	N.P.	N.P.	N.P.	N.P.	N.P.	N.P.	N.P.	1.45 (1.19-1.78)
CDT1	N.P.	N.P.	N.P.	N.P.	1.38 (1.17-1.63)	N.P.	N.P.	N.P.	N.P.	N.P.	N.P.
BUB1	N.P.	N.P.	N.P.	N.P.	1.47 (1.29-1.67)	N.P.	N.P.	N.P.	N.P.	N.P.	1.49 (1.22-1.82)
KIF18B	N.P.	N.P.	N.P.	N.P.	1.54 (1.38-1.71)	N.P.	N.P.	N.P.	N.P.	N.P.	1.41 (1.17-1.71)
ACACB	N.P.	N.P.	N.P.	N.P.	N.P.	N.P.	N.P.	N.P.	N.P.	N.P.	1.38 (1.17-1.62)
EPM2A	N.P.	N.P.	N.P.	N.P.	0.65 (0.53-0.8)	N.P.	N.P.	N.P.	N.P.	N.P.	1.52 (1.22-1.9)
RGN	N.P.	N.P.	N.P.	N.P.	0.75 (0.68-0.82)	N.P.	N.P.	N.P.	N.P.	N.P.	1.14 (1.04-1.26)
LYVE1	N.P.	N.P.	N.P.	N.P.	N.P.	N.P.	N.P.	N.P.	N.P.	N.P.	1.43 (1.23-1.65)
AOX1	N.P.	N.P.	N.P.	N.P.	0.9 (0.85-0.96)	N.P.	N.P.	N.P.	N.P.	N.P.	1.19 (1.08-1.3)
NR3C2	N.P.	N.P.	N.P.	N.P.	0.86 (0.79-0.93)	N.P.	N.P.	N.P.	N.P.	N.P.	N.P.
PGM5	N.P.	N.P.	N.P.	N.P.	0.85 (0.77-0.94)	N.P.	N.P.	N.P.	N.P.	N.P.	N.P.
GFRA2	N.P.	N.P.	N.P.	N.P.	N.P.	N.P.	N.P.	N.P.	N.P.	N.P.	1.18 (1.08-1.28)
FAXDC2	N.P.	N.P.	N.P.	N.P.	0.82 (0.73-0.93)	N.P.	N.P.	N.P.	N.P.	N.P.	N.P.
METTL7A	N.P.	N.P.	N.P.	N.P.	0.74 (0.67-0.81)	N.P.	N.P.	N.P.	N.P.	N.P.	N.P.

Table S20. Differentially expressed dp-lncRNA genes with prognostic value across cancer types using multivariate Cox proportional regression. *N.P.* refers that the given gene is non-prognostic on this cancer type

	Bladder	Breast	Colorectal	Kidney	Liver	Lung	Prostate	Stomach	Thyroid	Uterus
CATG00000017193.1	N.P.	N.P.	N.P.	1.19 (1.07-1.33)	N.P.	N.P.	N.P.	N.P.	N.P.	N.P.
SUGT1-DT	N.P.	N.P.	N.P.	1.36 (1.21-1.52)	N.P.	N.P.	N.P.	N.P.	N.P.	N.P.
CATG00000054098.1	N.P.	N.P.	N.P.	1.34 (1.2-1.5)	N.P.	N.P.	N.P.	N.P.	N.P.	N.P.
CATG00000087995.1	N.P.	N.P.	N.P.	1.22 (1.1-1.37)	N.P.	N.P.	N.P.	N.P.	N.P.	N.P.
CATG00000101363.1	N.P.	N.P.	N.P.	1.3 (1.16-1.45)	N.P.	N.P.	N.P.	N.P.	N.P.	1.29 (1.11-1.51)
PIK3IP1-DT	N.P.	N.P.	N.P.	1.28 (1.11-1.48)	N.P.	N.P.	N.P.	N.P.	N.P.	0.77 (0.65-0.91)
MORC2-AS1	N.P.	N.P.	N.P.	1.29 (1.17-1.43)	N.P.	N.P.	N.P.	N.P.	N.P.	N.P.
TMED2-DT	N.P.	N.P.	N.P.	1.28 (1.15-1.42)	N.P.	N.P.	N.P.	N.P.	N.P.	N.P.
SNHG1	N.P.	N.P.	N.P.	1.48 (1.26-1.73)	N.P.	N.P.	N.P.	N.P.	N.P.	1.42 (1.14-1.76)
MYG1-AS1	N.P.	N.P.	N.P.	N.P.	N.P.	N.P.	N.P.	N.P.	N.P.	1.39 (1.12-1.72)
ATP2A1-AS1	N.P.	N.P.	N.P.	1.26 (1.14-1.4)	N.P.	N.P.	N.P.	N.P.	N.P.	N.P.
NFE2L1-DT	N.P.	N.P.	N.P.	1.36 (1.21-1.52)	N.P.	N.P.	N.P.	N.P.	N.P.	N.P.
MRPL20-DT	N.P.	N.P.	N.P.	1.49 (1.29-1.72)	N.P.	N.P.	N.P.	N.P.	N.P.	1.46 (1.24-1.72)
KLF9-DT	N.P.	N.P.	N.P.	N.P.	N.P.	N.P.	N.P.	N.P.	N.P.	1.48 (1.21-1.83)
GAS1RR	N.P.	N.P.	N.P.	N.P.	N.P.	N.P.	N.P.	N.P.	N.P.	1.28 (1.15-1.42)
RAP2C-AS1	N.P.	N.P.	N.P.	0.76 (0.61-0.95)	N.P.	N.P.	N.P.	N.P.	N.P.	N.P.
CYP2U1-AS1	N.P.	N.P.	N.P.	1.22 (1.06-1.41)	N.P.	N.P.	N.P.	N.P.	N.P.	N.P.
SPCS3-AS1	N.P.	N.P.	N.P.	0.7 (0.55-0.88)	N.P.	N.P.	N.P.	N.P.	N.P.	N.P.
SETBP1-DT	N.P.	N.P.	N.P.	N.P.	N.P.	N.P.	N.P.	N.P.	N.P.	1.31 (1.13-1.53)
MAN2A1-DT	N.P.	N.P.	N.P.	0.75 (0.62-0.89)	N.P.	N.P.	N.P.	N.P.	N.P.	N.P.

Table S21. Differentially expressed ip-lncRNA genes with prognostic value across cancer types using multivariate Cox proportional regression. *N.P.* refers that the given gene is non-prognostic on this cancer type

	Bladder	Breast	Colorectal	Head and Neck	Kidney	Liver	Lung	Prostate	Stomach	Thyroid	Uterus
SNHG17	N.P.	N.P.	N.P.	N.P.	1.72 (1.46-2.01)	N.P.	N.P.	N.P.	N.P.	N.P.	N.P.
LINC01311	N.P.	N.P.	N.P.	N.P.	1.27 (1.14-1.43)	N.P.	N.P.	N.P.	N.P.	N.P.	N.P.
MIR99AHG	N.P.	N.P.	N.P.	N.P.	N.P.	N.P.	N.P.	N.P.	N.P.	N.P.	1.21 (1.09-1.35)
LINC00667	N.P.	N.P.	N.P.	N.P.	N.P.	N.P.	N.P.	N.P.	N.P.	N.P.	1.2 (1.08-1.33)

Table S22. Differentially expressed e-lncRNA genes with prognostic value across cancer types using multivariate Cox proportional regression. *N.P.* refers that the given gene is non-prognostic on this cancer type

	Bladder	Breast	Colorectal	Head and Neck	Kidney	Liver	Lung	Prostate	Stomach	Thyroid	Uterus
CATG00000107122.1	0.82 (0.70-0.97)	N.P.	N.P.	N.P.	1.27 (1.15-1.41)	N.P.	N.P.	N.P.	N.P.	N.P.	N.P.

Table S23. List of new attributed HUGO Symbol.

Ensembl ID	NCBI ID	FANTOM-CAT ID	Ensembl Release 99	HGNC symbol	HGNC name
ENSG00000273723	NA	CATG00000017193.1	AL139089.1	SUGT1-DT	SUGT1 divergent transcript
ENSG00000257605	NA	RP11-680A11.5	AC073611.1	MYG1-AS1	MYG1 antisense RNA 1
ENSG00000258384	NA	AC068831.6	AC068831.2	RCCD1-AS1	RCCD1 and UNC45A antisense RNA 1
ENSG00000263412	NA	RP5-890E16.2	AC004477.1	NFE2L1-DT	NFE2L1 divergent transcript
ENSG00000270195	NA	RP11-572O17.1	AC016773.2	SLBP-DT	SLBP divergent transcript
ENSG00000272455	NA	RP4-758J18.13	AL391244.2	MRPL20-DT	MRPL20 divergent transcript
NA	101927086	CATG00000105517.1	NA	KLF9-DT	KLF9 divergent transcript
ENSG00000225793	100506804	RP1-234P15.4	AL080250.1	TMEM30A-DT	TMEM30A divergent transcript
ENSG00000235652	100507557	RP11-545I5.3	AL356599.1	FBXO30-DT	FBXO30 divergent transcript
ENSG00000245293	101929595	RP11-286E11.1	AC096564.1	CYP2U1-AS1	CYP2U1 and SGMS2 antisense RNA 1
ENSG00000248980	NA	RP11-87F15.2	AC019163.1	SPCS3-AS1	SPCS3 antisense RNA 1
ENSG00000267414	101927943	RP11-456K23.1	AC120049.1	SETBP1-DT	SETBP1 divergent transcript
ENSG00000271849	NA	CTC-332L22.1	AC012603.1	MAN2A1-DT	MAN2A1 divergent transcript
ENSG00000272686	NA	RP11-390E23.6	AC006333.1	WASL-DT	WASL divergent transcript
ENSG00000231246	105378909	RP5-965F6.2	AL445426.1	LINC02884	long intergenic non-protein coding RNA 2884
ENSG00000272666	NA	CTA-384D8.35	U62317.1	KLHDC7B-DT	KLHDC7B divergent transcript
ENSG00000255958	NA	RP11-656E20.5	AC115676.1	GABARAPL1-AS1	GABARAPL1 antisense RNA 1

Table S24. List of features used in the multivariate Cox regression analysis.

Cancer type	Features
Breast	stage, age, Estrogen/Progesterone Receptor, HER2 status
Bile	stage, sex, age, CA19
Bladder	stage, sex, age
Colorectal	stage, sex, age, CEA
Esophagus	stage, sex, age, histological class
Head and Neck	stage, sex, age, perineural invasion presence, packs smoked
Kidney	stage, sex, age, morphology
Liver	stage, sex, age
Lung	stage, sex, age, histology, packs smoked
Stomach	stage, sex, age, histology
Thyroid	stage, sex, age, extrathyroid carcinoma extension
Prostate	age, gleason
Uterus	age, tumor grade

Supplementary Figures

List of Figures

S1 Tissue specific expression in GTEx. 17

S2 Coding and non-coding RNAs in prostate cancer 18

S3 Distribution of the percentage of samples in GTEx expressing each gene 19

S4 Distribution of genes not expressed in GTEx by RNA class 20

S5 KM curve for enhancer 22 21

S6 KM curves prognostic non-coding RNAs 22

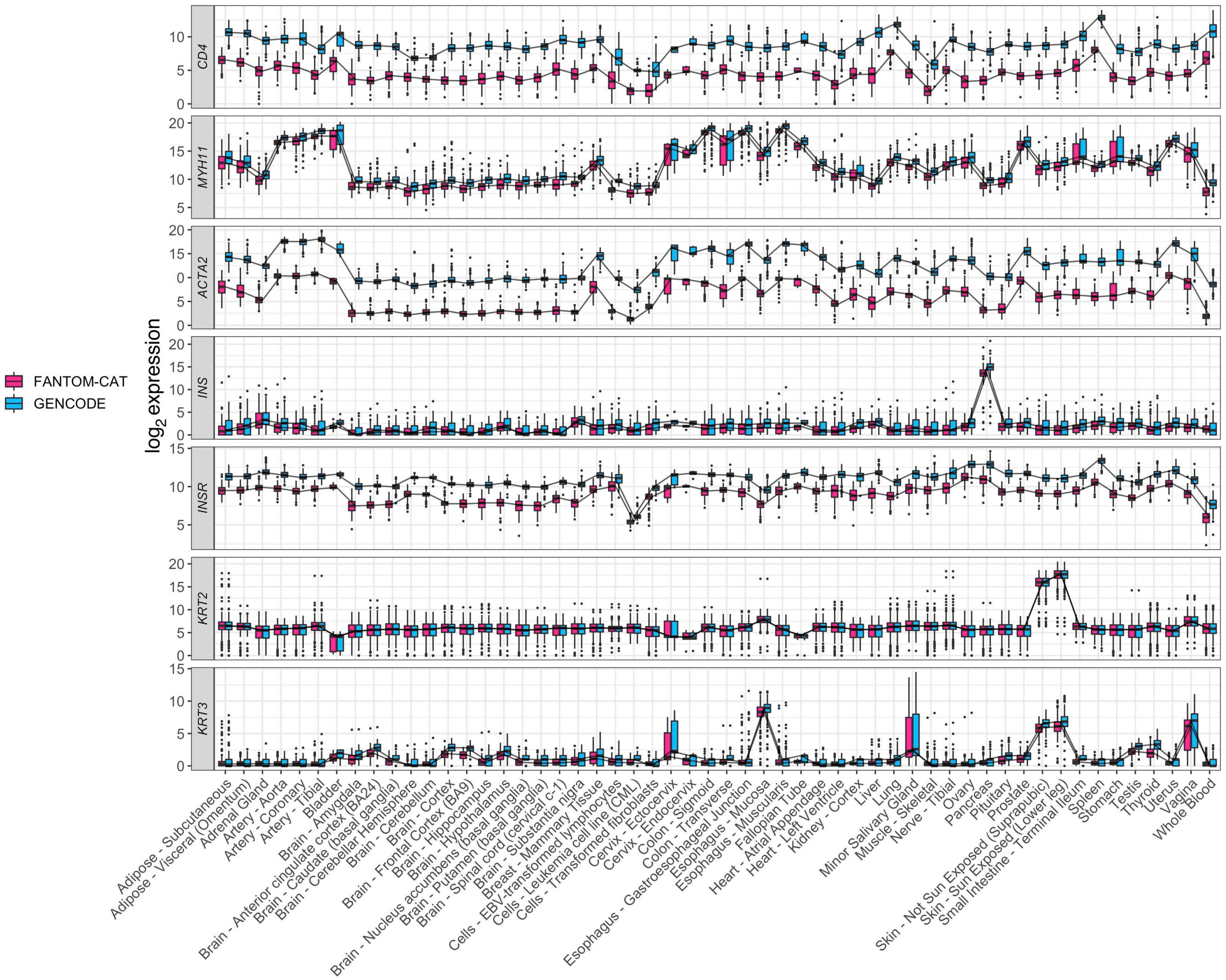

Figure S1. **Figure S1. Tissue specific expression in GTEx.** $\log_2$ expression for tissue specific genes in GTEx data stratify by tissue type using `FC-R2` and GENCODE based quantification. Expression profiles are highly correlated and expressed consistently in the expected tissue types. *CD4* is most expressed in spleen, *MYH11* and *ACTA2* in tissues consisted of muscle cells, *INS* in pancreas and its receptor *INSR* globally, *KRT2* and *KRT3* in skin.

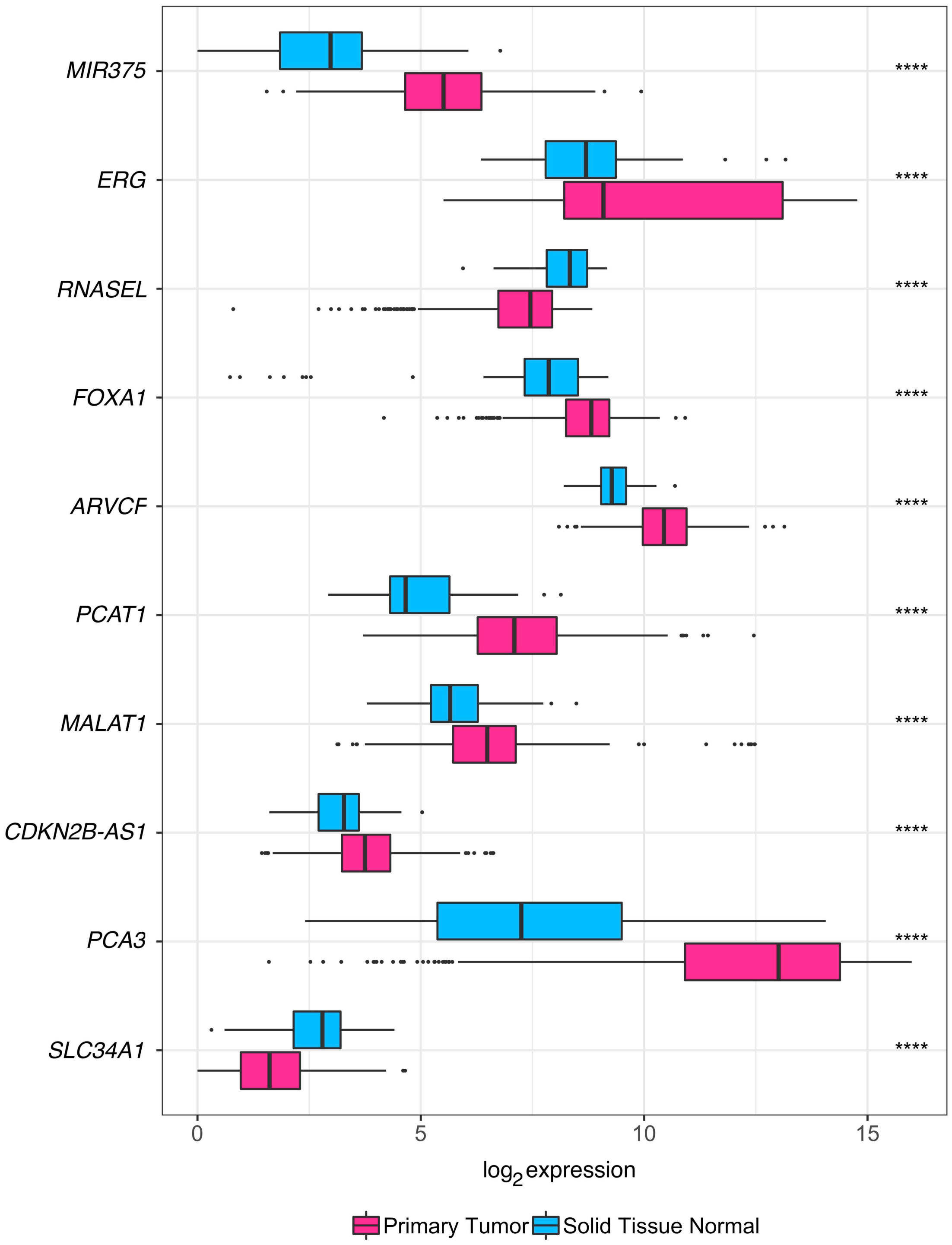

Figure S2. Figure S2. Coding and non-coding RNAs in prostate cancer. Expression levels for coding and non-coding RNAs known to be involved in prostate cancer. Results are stratified by sample type, pink is used for normal primary tissue, while light blue for primary cancers. The stars indicate significance from the moderated t-test ($FDR < 0.0001$).

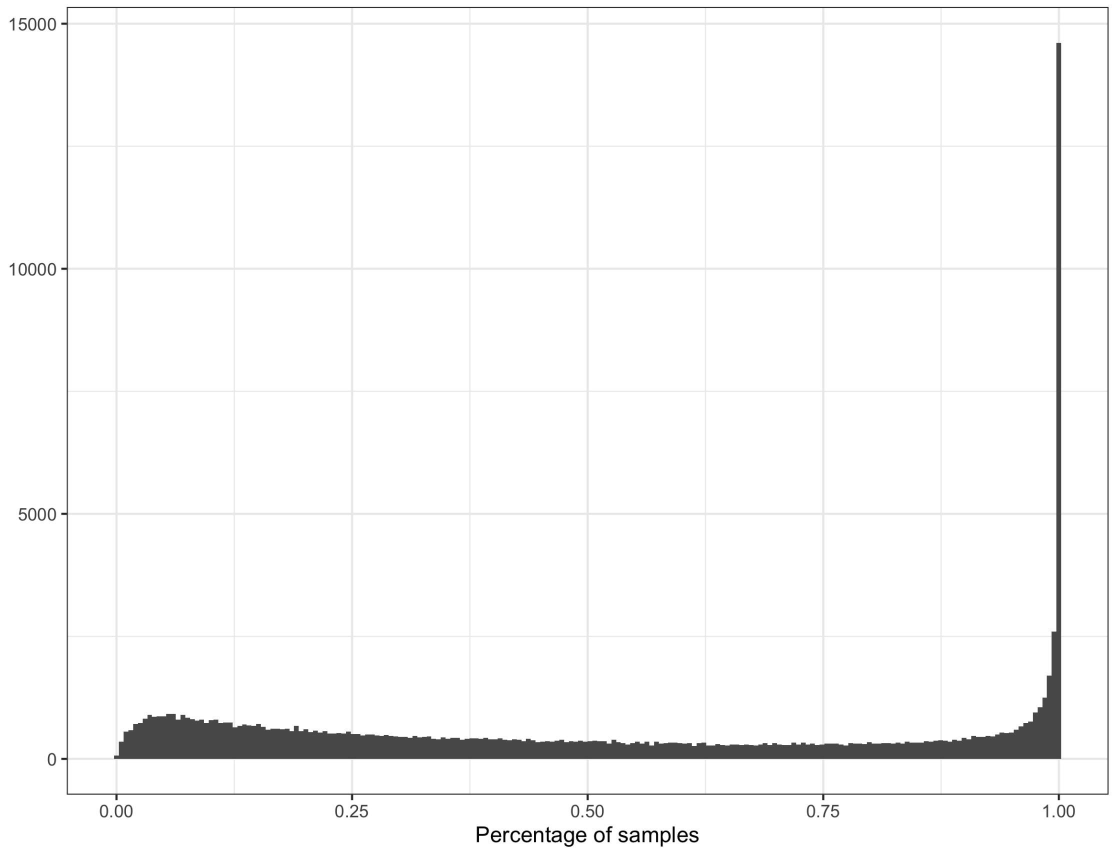

Figure S3. Figure S3. Distribution of the percentage of samples in GTEx expressing each gene. Genes with TPM < 0.01 for over 99% of the samples were considered not expressed. 681 genes out of 109,869 comprised in the resource were not expressed in any samples.

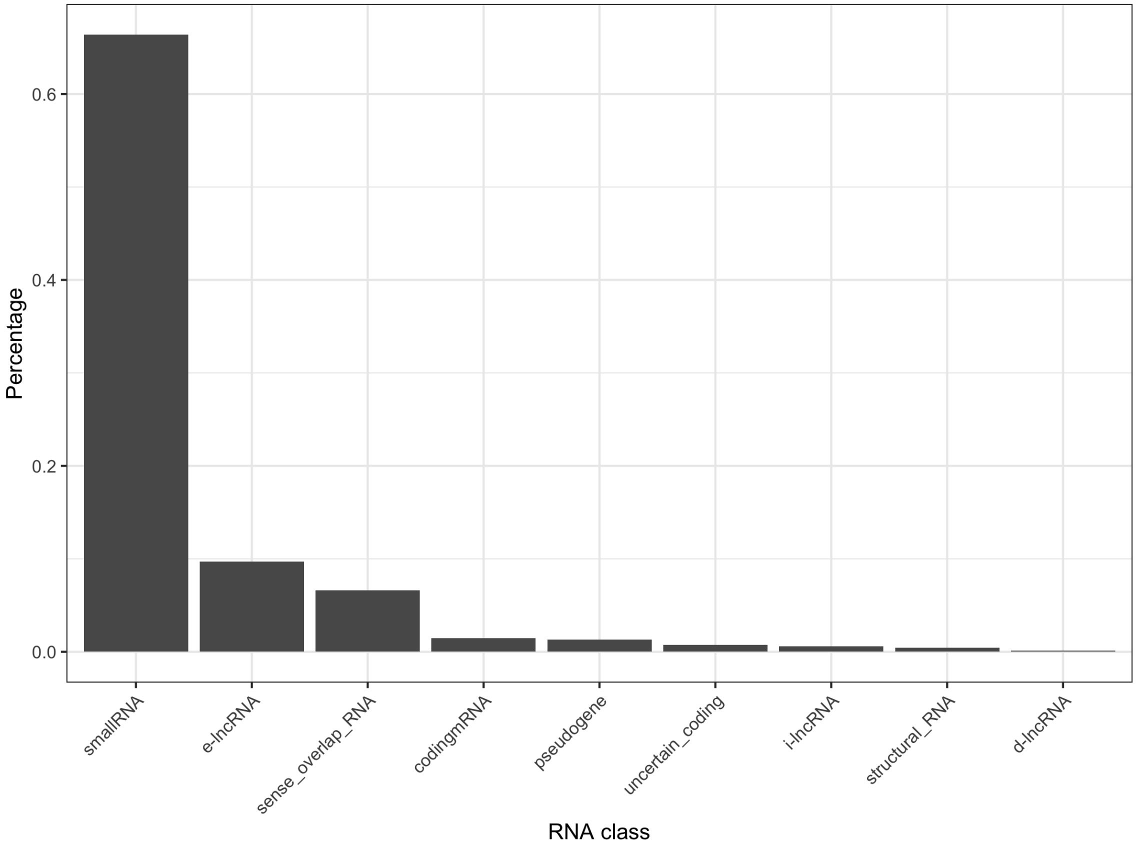

Figure S4. Figure S4. Distribution of genes not expressed in GTEx by RNA class. The genes showing a TPM value below 0.01 in at least 99% of the GTEx samples were considered not expressed. The breakdown by RNA class for the resulting 681 not expressed genes is shown.

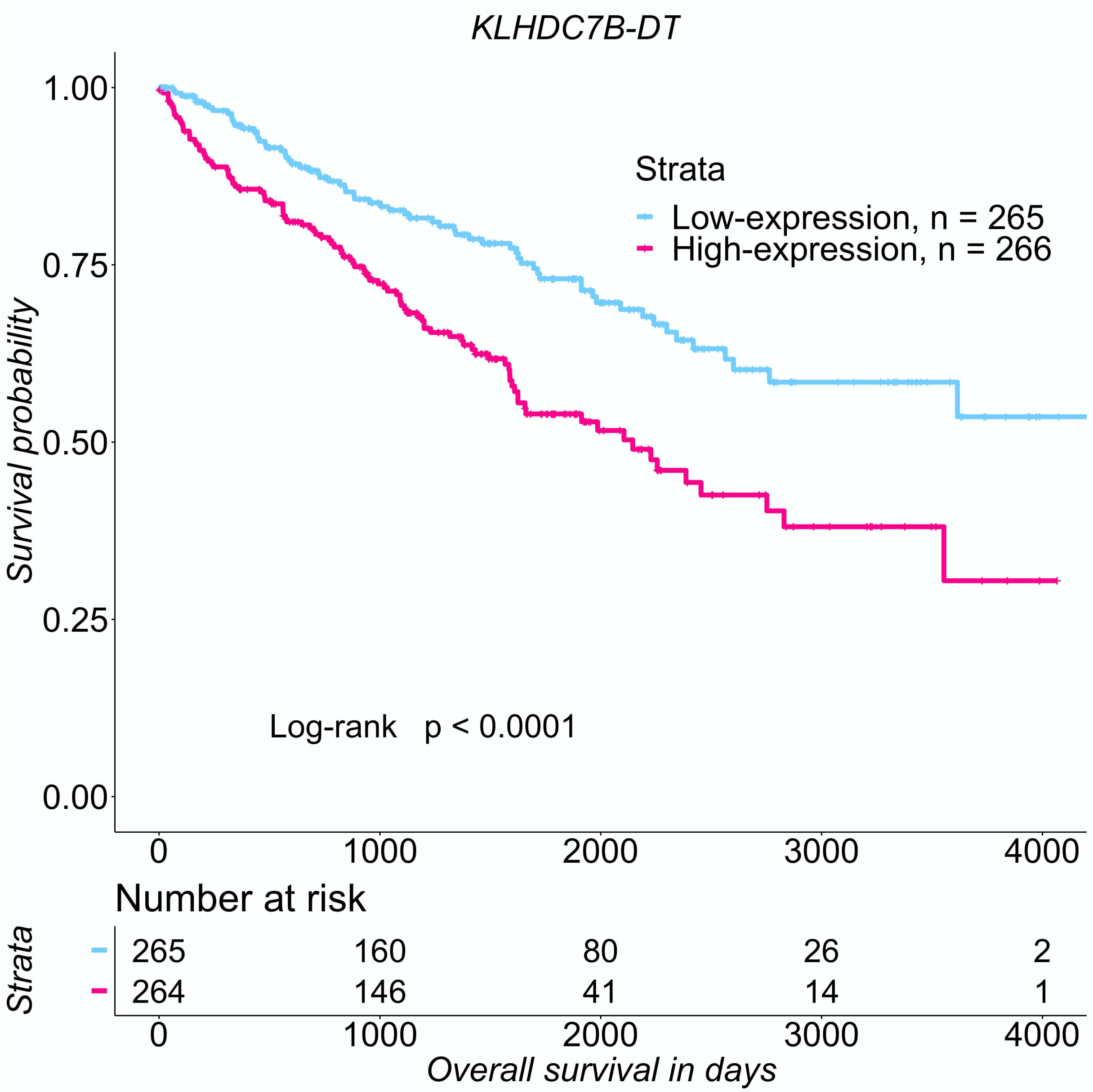

Figure S5. **Figure S5. KM curve for enhancer 22.** Kaplan-Meier survival curve depicting gene *KLHDC7B-DT* - enhancer22 in Chen et al. (2018)) groups split by median expression level for kidney clear cell renal cell carcinoma (KIRC) cases. Statistical significances were assessed using log-rank test.

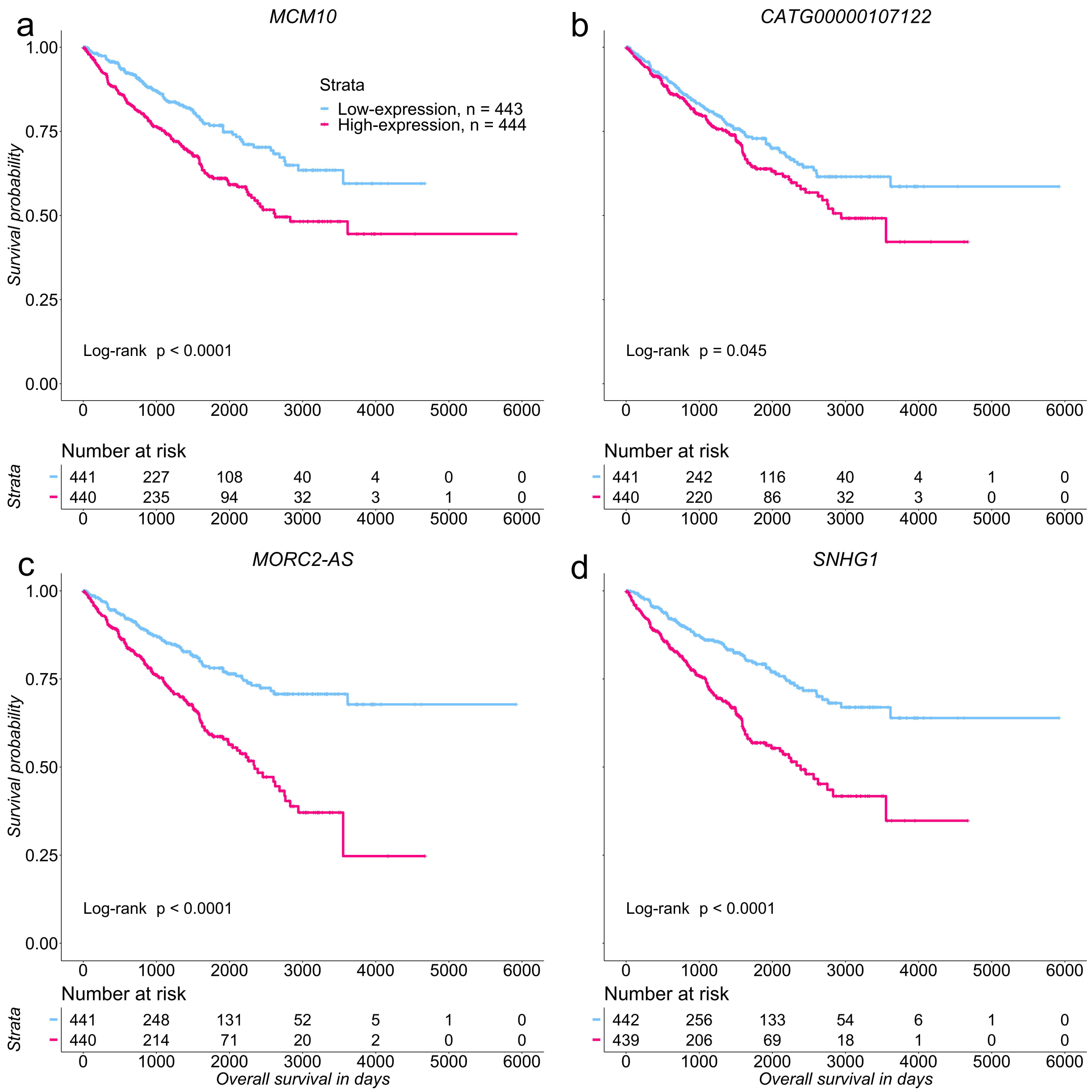

Figure S6. Figure S6. Kaplan-Meier survival curves depicting four selected differentially expressed genes holding prognostic value in kidney cancers. a. mRNA gene *MCM10 (ENSG00000065328)*, **b.** e-lncRNA gene *CATG00000107122*, unique to *FANTOM-CAT*, **c.** dp-lncRNA gene *MORC2-AS1 (ENSG00000235989)*, **d.** ip-lncRNA gene *SNHG17 (ENSG00000196756)*. Low- and high-expression groups were split by median expression level for each gene. Statistical significances were assessed using log-rank test.

Supplementary Methods

Data and pre-processing.

We obtained files containing coordinates of updated gene models of FANTOM-CAT permissive set from the ongoing FANTOM6 consortium. These files containing coordinates for 709,176 transcript models were imported into an R session and used to create a Genomic Range object with the GenomicRanges package (Lawrence et al. 2013) of all exons coordinates. Given the unstranded nature of *recount2* we opted to remove overlapping exons belonging to different gene models to avoid over-quantification of these regions. To avoid losing strand information from annotation we first split exons coordinates by strand into two objects. We then used the `disjoin` function from Genomic Ranges package to generate disjoint segments in each strand, and then across strands (see Figure 5 in the main paper). Each segment coordinates were then assigned to the corresponding overlapping gene model. Segments that were assigned to more than one gene were discarded. The expression values for each segment was then quantified using *recount.bwtool* (Ellis et al. 2018) (code at `https://github.com/LieberInstitute/marchionni_projects`). The resulting expression quantifications were processed to generate `RangedSummarizedExperiment` objects compatible with the *recount2* framework (Collado-Torres et al. 2017b,a) (code at `https://github.com/eddieimada/fcr2`). The expression values for each segment weer added up for gene model, resulting in the final object containing expressions at gene level, which are distributed through the *recount* package and the *recount2* website.

Correlation with other studies.

Due to the decision of removing segments overlapping with more than one gene model, we further investigated if removal of these segments caused significant impact on expression levels. To achieve that we compared our GTEx counts tables to the published GTEx counts from *recount2*. The version 2 of the gene counts for the GTEx samples were downloaded from the recount website (`https://jhubiostatistics.shinyapps.io/recount/`). Next, we scaled each object to a 40M depth using the `scale_counts` function from recount package. After scalling, we obtained the intersection of the genes across both objects and computed the Pearson correlation for each gene. We further selected a few tissue markers to evaluate expression specificity across tissue types.

Expression specificity of tissue facets.

To analyze the expression level and specificity of each gene, we first scaled GTEx data to 40M depth using the `scale_counts` function from recount package. Genes were then stratified by RNA class (i.e. mRNA, e-lncRNA, dp-lncRNA, ip-lncRNA) and grouped by tissue type (n = 54 facets). The expression level for each gene was represented by the maximum transcripts per million (TPM) of all samples within a facet. The expression specificity was calculated as the empirical entropy of the mean expression values of each facet divide by the log_2 of the number of facets, as follows

$$SPECIFICITY = 1 - (entropy(X)/log_2(N))$$

Where X is a vector of sample-average values for a given gene over all facets and N is the number of facets. The 99.99 percent confidence intervals for the expression of each category by facet were calculated based on TPM values. Genes with a TPM greater

than 0.01 were considered expressed.

Identification of differentially expressed genes.

To perform differential gene expression analysis across cancer types we relied on TCGA data scaled to 40M depth using the `scale_counts` function from recount. We split each cancer dataset by RNA class (coding mRNA, intergenic promoter lncRNA, divergent promoter lncRNA and enhancer lncRNA) and removed all metastatic samples prior analysis. Each RNA class was treated independently.

The design matrices were created from a factor with two levels (Primary Tumor and Normal Tissue) by setting the normal tissues as the intercept. For each RNA class we removed genes with low expression (< 5 counts) in more than 1/3 of the total samples in each cancer type. After filtering, we normalized raw libraries sizes using method TMM with `calcNormFactors` from edgeR package. After normalization, we transformed the count data to log_2-counts per million (logCPM), and estimated the mean-variance relationship to compute appropriate observation-level weights using `voom`.

Finally, we run surrogate variable analysis (SVA) (Leek and Storey 2007) using the permutation procedure proposed by Buja and Eyuboglu (Buja and Eyuboglu 1992) and added the first three most variable coefficients as covariates in our design matrix. A generalized linear model approach coupled with empirical Bayes standard errors (Smyth 2004) was then used for identifying differentially expressed genes between the samples. Correction for multiple testing was performed across RNA classes by merging the resulting p-values for each cancer type and applying the Benjamini-Hochberg method (Benjamini and Hochberg 1995). Overlapping between DEG and GWAS SNPs was performed using the genes coordinates and SNPs obtained from GWAS Catalog (Buniello et al. 2018) with the GenomicRanges package (Lawrence et al. 2013).

Confirming prognostic enhancers.

Univariate and Multivariate Cox proportional regression were performed in four RNA subtypes (22106 mRNAs, 17,404 e-lncRNAs, 6,204 dp-lncRNAs, and 1,948 ip-lncRNAs) available through `FC-R2` as predictors on each of the 13 TCGA cancer types with available survival follow-up. The gene expressions were obtained across cancer types on TCGA data scaled to 40M depth using the `scale_counts` function from *recount2* and logged. We split each cancer dataset by RNA class (coding mRNA, intergenic promoter lncRNA, divergent promoter lncRNA and enhancer lncRNA) using only primary tumor samples gene expression. Some patients had more than one sample, in such cases, the first sample was chosen for the gene expression levels. Each RNA class was treated independently. Survival analysis including Cox proportional regression was done using R *survival* package. Survival data (follow-up and vital status) available in the phenotype data derived from *recount2 expressionSets* were used to create survival objects for each of the 13 cancer types. Cox proportional regression was done for each cancer type using one gene at a time (univariate regression) grouped on each of the four RNA categories surveyed. Genes were considered predictive if its FDR were equal or less than 0.05, using Benjamini-Hochberg (Benjamini and Hochberg 1995) correction. The correction was done within a cancer type grouping all genes by RNA type (i.e. corrected for 22,106 genes if mRNA were surveyed). This procedure yielded the predictive genes list broken-down in the four RNA categories reported. For reporting differentially expressed genes (DEG) among all cancer types that portrait predictive value, the DEG lists were surveyed on each of the significant prognostic genes lists generated during the univariate Cox analysis by cancer types and summary tables reporting the genes predictive potential were prepared. The same procedure above was followed for the multivariate Cox proportional regression analysis using relevant biological features for each cancer type (see Supplemebtary Table

S23). Each feature was considered on a case by case basis taking in account the amount of missing data and correlation with other features. Kaplan-Meier curves were done using *survminer* R package (Kassambara and Kosinski 2018). Groups were defined using median gene expression level and significance were assessed by log-rank test (P-value < 0.05).

Supplementary data from Chen et al. (Chen et al. 2018) containing enhancers position and prognostic potential were obtained from the original publication supplementary material. Liftover to hg38 genome assembly was performed to match `FC-R2` coordinates in order to compare both resources. Prognostic genes list provided in Chen's paper were compared to prognostic genes obtained using `FC-R2`.
